# Comprehensive multi‐omics mapping of immune perturbations in autism spectrum disorder

**DOI:** 10.1002/ctm2.70552

**Published:** 2025-12-12

**Authors:** Chun Yan, Fangmei Feng, Chaoting Lan, Gang Luo, Xiaotao Jiang, Huijuan Wang, Yinchun Chen, Yuling Yang, Liangqiong Deng, Xiaoli Huang, Yuxin Wu, Wenxiong Chen, Yufeng Liu

**Affiliations:** ^1^ Liuzhou Hospital of Guangzhou Women and Children's Medical Center Liuzhou Guangxi China; ^2^ Center for Medical Research on Innovation and Translation Guangzhou First People's Hospital The Second Affiliated Hospital of South China University of Technology Guangzhou Guangdong China; ^3^ Department of Neuroelectrophysiology, Guangzhou Women and Children's Medical Center Guangzhou Medical University Guangzhou Guangdong China; ^4^ Jianghai Street Community Health Service Center Guangzhou Guangdong China; ^5^ First Clinical Medical College Guangzhou University of Chinese Medicine Guangzhou Guangdong China; ^6^ Department of Hematology, The Second Affiliated Hospital, School of Medicine South China University of Technology Guangzhou Guangdong China; ^7^ Department of Hematology Guangzhou First People's Hospital Guangzhou Guangdong China; ^8^ Department of Children Healthcare Liuzhou Hospital of Guangzhou Women and Children's Medical Center Liuzhou Guangxi China; ^9^ Department of Neurology, Liuzhou Hospital of Guangzhou Women and Children's Medical Center Liuzhou Key Laboratory of Pediatric Epilepsy Prevention and Treatment Liuzhou Guangxi China; ^10^ Department of Rehabilitation, Guangzhou Women and Children's Medical Center Guangzhou Medical University Guangzhou Guangdong China; ^11^ Department of Neurology, Guangzhou Women and Children's Medical Center Guangzhou Medical University Guangzhou Guangdong China; ^12^ Department of Behavioral Development, Guangzhou Women and Children's Medical Center Guangzhou Medical University Guangzhou Guangdong China

**Keywords:** autism spectrum disorder, biomarkers, immune dysregulation, multi‐omics analysis

## Abstract

**Background:**

Autism spectrum disorder (ASD) is increasingly recognized as a neurodevelopmental condition with systemic immunological involvement, yet the underlying immune mechanisms remain incompletely defined.

**Aims:**

To delineate the peripheral immune landscape in ASD using integrated multi‐omics profiling and to determine how immune and immunometabolic alterations relate to clinical severity.

**Materials & Methods:**

Circulating immune cells from individuals with ASD were profiled using multicolor flow cytometry, single‐cell RNA sequencing, and bulk RNA sequencing. Plasma proteomic and metabolomic analyses were performed to identify immune‐related and metabolic biomarkers. Immune features were evaluated for associations with clinical severity measures.

**Results:**

Multi‐omics profiling revealed marked immune dysregulation in ASD, with significant shifts in immune cell subsets and inflammatory signatures that correlated with clinical severity. T cell abnormalities included reduced frequencies and a skewed Th1/Th2 balance, consistent with a chronic inflammatory milieu. Natural killer (NK) cells showed increased activation but impaired cytotoxic capacity, accompanied by expansion of an atypical NK subset. Myeloid‐derived suppressor cells (MDSCs) and hyperinflammatory CD56+ monocytes were elevated. Transcriptomic analyses corroborated broad immune activation, prominently implicating interferon‐driven and antiviral signaling pathways. Plasma metabolomics and proteomics further indicated disruptions in purine metabolism and oxidative phosphorylation, alongside increased inflammatory markers, which were significantly associated with symptom severity.

**Discussion:**

These findings support a systemic immunometabolic framework in ASD characterized by concurrent immune activation and altered myeloid/NK cell states, providing mechanistic context for peripheral biomarkers linked to clinical phenotype.

**Conclusion:**

Integrated multi‐omics profiling identifies robust peripheral immune and metabolic disturbances in ASD. The dysregulated immune subsets, activated immune pathways, and plasma biomarker signatures highlight potential avenues for biomarker‐driven stratification and immune‐targeted therapeutic development in ASD.

**Key points:**

- T cell dysregulation, NK cell impairment, and myeloid expansion indicate a chronic inflammatory state and immune exhaustion phenotype associated with ASD severity.
- Plasma metabolomic and proteomic alterations, including disrupted oxidative phosphorylation and elevated inflammatory markers, correlate with ASD severity and highlight potential biomarkers.
- Multi‐omics profiling links peripheral immune dysregulation to neurodevelopmental abnormalities, providing a framework for immune‐targeted ASD interventions.

## Graphical abstract

• Integrated multi‐omics profiling reveals systemic immune dysregulation in ASD, marked by an expansion of atypical immune cell subsets and activation of immune‐related pathways.

• Significant alterations in peripheral immune cell populations are observed, including dysregulated NK cells, expanded CD56+ monocytes, and elevated MDSCs, and these changes correlate with clinical severity measures in ASD.

• Concomitant metabolic and inflammatory disturbances further define an immunometabolic framework for ASD‐related neuroinflammation.

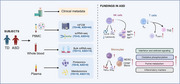

## 1 INTRODUCTION

Autism spectrum disorder (ASD) is a multifaceted neurodevelopmental condition characterized by impairments in social interaction, deficits in communication and the presence of repetitive patterns of behaviour.^1^ Although genetic,^2^ neurological,^3^ and environmental factors^4^ have been extensively documented, the immunological dimension of ASD is comparatively understudied. Emerging evidence indicates that immune dysfunction may contribute to ASD pathophysiology, potentially through mechanisms involving neuroinflammation or disrupted neuroimmune crosstalk.^5^, ^6^ However, the immune system's inherent complexity, encompassing diverse cell types, cytokine networks and signalling pathways, continues to obscure the precise nature and functional implications of these alterations in ASD.

Existing research has primarily focused on isolated immune components. In the innate immune system, aberrant activation of monocytes and dendritic cells has been observed,^7^, ^8^ together with elevated pro‐inflammatory cytokines^9^ and overexpression of immune regulatory genes.^10^ Moreover, macrophages derived from individuals with ASD exhibited increased TNF‐α secretion.^11^ In the adaptive immune system, dysregulated T cell activity has been documented, exhibiting alterations in cellular function, inflammatory phenotypes and modifications in cytokine secretion and intracellular signalling pathways.^12^ Additionally, natural killer (NK) cells were frequently elevated in number but displayed reduced cytotoxic capacity.^13^, ^14^ Beyond immune cells, metabolomic studies have identified dysregulated metabolites associated with purine metabolism, oxidative stress and mitochondrial dysfunction in ASD.^15^, ^16^

Although these findings collectively point to pervasive immune dysregulation in ASD, current studies face several methodological challenges: small and heterogeneous cohorts, limited analyte coverage and inconsistent protocols that hinder reproducibility and cross‐study comparisons. Moreover, immune alterations in ASD likely result from an intricate interaction between genetic susceptibility^17^ and environmental factors.^18^, ^19^, ^20^ These factors underscore the need for an integrated, multidisciplinary framework that combines immunology, neuroscience, genetics and psychiatry to unravel immune–neurodevelopmental interactions and inform targeted interventions.

In this study, we employed multicolour flow cytometry (mFCM) and single‐cell RNA sequencing (scRNA‐seq) to characterize circulating immune cells and utilized bulk RNA sequencing (RNA‐seq) to assess immune transcriptional alterations in ASD whole blood. Plasma multi‐omics analyses were further applied to profile protein and metabolite features. By integrating these multi‐omics approaches, we aim to delineate alterations and interrelationships among peripheral immune components in ASD and to explore their potential roles in disease pathogenesis and progression.

## 2 RESULTS

### 2.1 Overview of the multi‐omic characterization of immune responses in ASD patients

**FIGURE 1:**
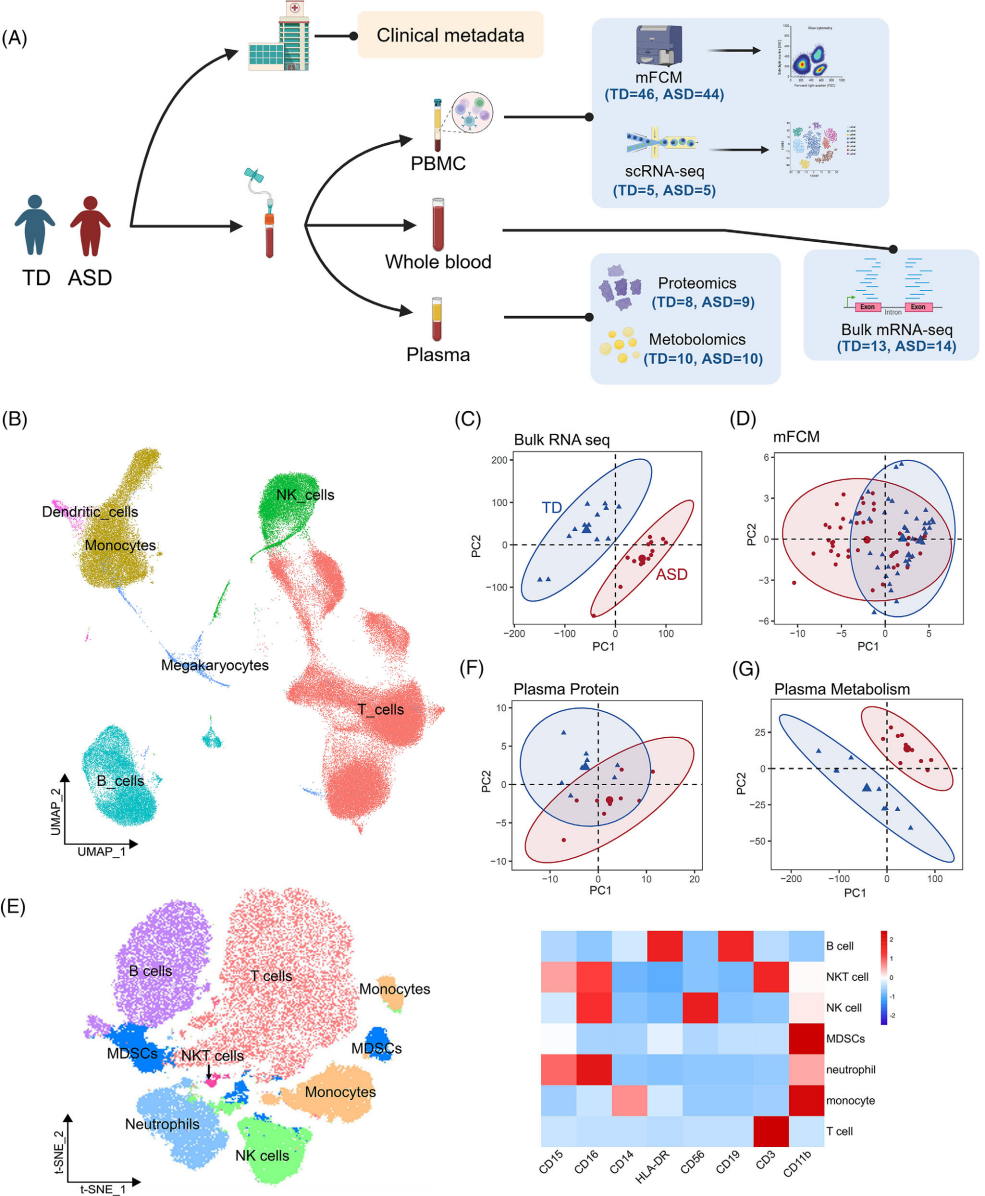
Overview of the immune cells and plasma multi‐omics characterization in autism spectrum disorder (ASD) patients. (A) Graphical overview of the study cohorts. (B) Uniform manifold approximation and projection (UMAP) embedding of six major cell lineages (colours) in peripheral blood mononuclear cells (PBMC) single‐cell RNA sequencing (scRNA‐seq) profiles from five ASD patients and five typically developing (TD) individuals. **(C** and D) Principal component analysis (PCA) of bulk RNA sequencing (RNA‐seq) (C) and multicolour flow cytometry (mFCM) data (D). Each dot represents one individual sample, coloured red for ASD and blue for TD. (E) *t*‐SNE analysis of mFCM data illustrating the distribution of subsets in PBMCs from ASD patients and TD individuals. Left: Each dot represents a single cell, coloured based on sequential bivariate gating. Right: Heatmap showing the relative expression of representative markers across major subsets. (F–G) PCA of plasma proteomics (F) and metabolomics (G) data. Each dot represents one plasma sample, with ASD samples in red and TD samples in blue.

We collected peripheral blood samples from 63 children with newly diagnosed ASD. In parallel, 64 typically developing (TD) controls were enrolled, matched by age and gender (Figure S1A, Tables S1 and S2). Detailed clinical indicators of ASD and blood routine results were analysed, showing significant positive correlations between white blood cell and lymphocyte counts with developmental quotient (DQ)/intelligence quotient (IQ), and significant negative correlations with ASD development scales (Figure S1B,C, Tables S1 and S3). These findings underscore the potential association between immune cells and ASD development. Consequently, we conducted a comprehensive analysis of clinical metadata, immune cells, transcriptional regulation, proteins and metabolomic characteristics to elucidate disease features (Figure 1A). The peripheral blood mononuclear cells (PBMC) scRNA‐seq data were mapped to a two‐dimensional (2D) projection using uniform manifold approximation and projection (UMAP^21^; Figure 1B), resolving six major cell lineages based on canonical markers (Figure S2B).^22^ All clusters contained cells from multiple samples, indicating no significant batch effects (Figure S2C,D). To comprehensively explore alterations in immune cell composition in ASD, we integrated bulk RNA‐seq data with scRNA‐seq data for mutual validation. Principal component analysis (PCA) of bulk RNA‐seq data distinctly segregated ASD patients from TD individuals, indicating significant transcriptional differences (Figure 1C). We identified 377 upregulated and 713 downregulated differentially expressed genes (DEGs) in ASD from bulk RNA‐seq profiles (Figure S2E, Table S4). Mapping these DEGs to scRNA‐seq cell lineages revealed enrichment of upregulated DEGs in T cells and monocytes, whereas downregulated genes were primarily enriched in NK cells and monocytes (Figure S2F,G). These differential expression patterns in ASD bulk transcriptome may reflect differences in cellular composition. To further investigate these variations, particularly in T cells, NK cells and monocytes, we employed mFCM to examine peripheral immune cell phenotypes in ASD. Results of the mFCM and antibody details are displayed in Tables S5 and S6, and the gating strategy is available in Figure S3. PCA clustering confirmed differences in immune cell profiles between ASD and TD individuals, with *t*‐distributed stochastic neighbour embedding (*t*‐SNE) analysis showing separate immune cell compositions (Figure 1D,E). Analysed plasma proteins and metabolites are listed in Tables S7 and S8. PCA of 92 plasma proteins and 5491 plasma metabolites demonstrated significant differences between ASD and TD individuals (Figure 1F,G). The observed multi‐omics variances prompted us to further investigate the transcriptional, proteomic and metabolic differences within each major cell subpopulation.

### 2.2 Altered T cell phenotypic composition and immune activation in ASD

**FIGURE 2:**
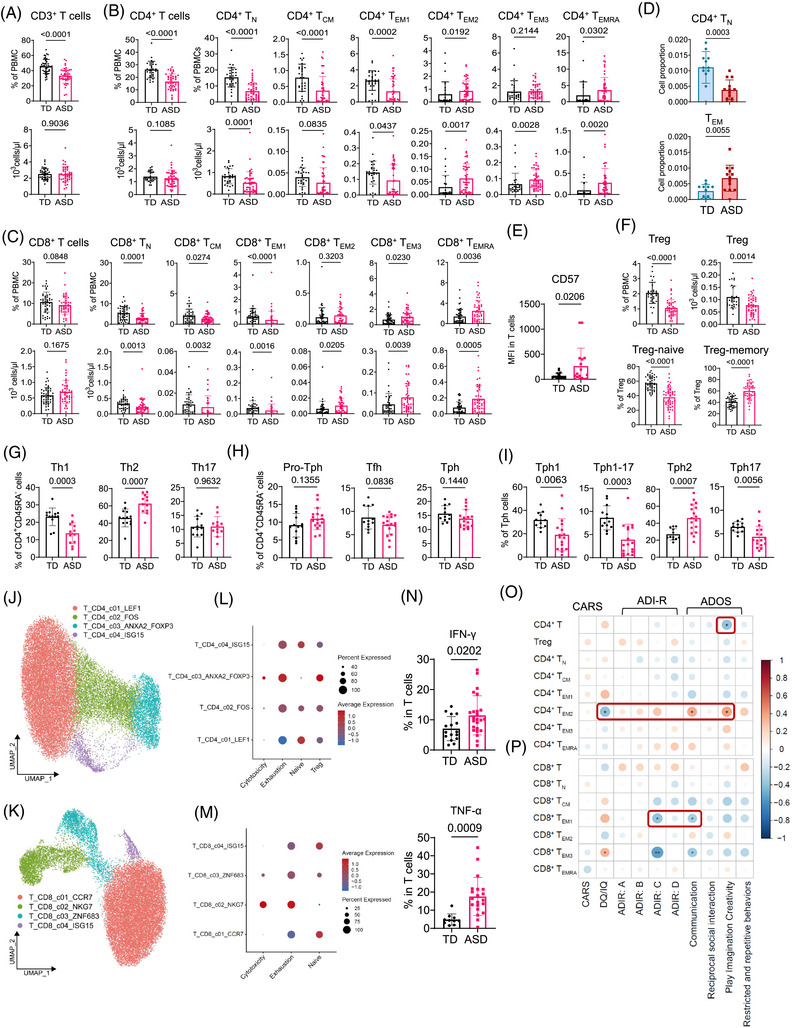
T cell heterogeneity in autism spectrum disorder (ASD) and its association with clinical metadata. (A–C) Proportions and absolute counts of CD3^+^ T cells (A), CD4^+^ T cell subsets (B) and CD8^+^ T cell subsets (C) in ASD patients (*n* = 44) and typically developing (TD) individuals (*n* = 46). The gating strategy is detailed in Figure S3. (D) Boxplots illustrating the differences in proportions of CD4^+^ T_N_ and T_EM_ cells between ASD patients (*n* = 14) and TD individuals (*n* = 13) in the bulk RNA sequencing (RNA‐seq) data, which was deconvolved by the ImmuneCellAI database. (E) Surface expression of CD57 in T cells from ASD patients (*n* = 17) and TD individuals (*n* = 21). (F) Proportions and absolute counts of T regulatory (Treg) cells and its subsets in ASD patients (*n* = 44) and TD individuals (*n* = 46). (G–I) Proportions of T helper (Th) subsets in CD4^+^ T cells (G); T follicular helper (Tfh), T peripheral helper (Tph) and pro‐Tph cells in CD45RA^−^ memory CD4^+^ T cells (H); and Tph subsets in Tph cells (I) isolated from ASD patients (*n* = 12–15) and TD individuals (*n* = 12–13). (J and K) Uniform manifold approximation and projection (UMAP) embedding of 27 359 CD4^+^ T cells (J) and 23 465 CD8^+^ T cells (K) from all single‐cell RNA sequencing (scRNA‐seq) samples. Each point represents one cell. Cells coloured according to the cell cluster. (L and M) Dot plot depicting average expression levels and percentages of cells expressing cytotoxic, exhaustion, naïve and T regulatory (Treg) signature scores in CD4^+^ T cell (L) and CD8^+^ T cell (M) subsets from all scRNA‐seq samples. (N) Percentage of IFN‐γ and TNF‐α positive cells in T cells from ASD patients (*n* = 24) and TD individuals (*n* = 16), as determined by multicolour flow cytometry (mFCM). (O and P) Correlation matrix between the frequencies of T cell subsets and clinical diagnostic scale scores. The circle size corresponds to the absolute value of the Spearman correlation coefficient, with red (blue) colour indicating a positive (negative) correlation. ^*^ *p* < .05, ^**^ *p* < .01. For A–I and N, data are presented as mean ± standard deviation (SD), and the two‐tailed Wilcoxon rank sum test (D) and two‐tailed unpaired *t*‐tests (A–C, E–I and N) were applied. ADI‐R, Autism Diagnostic Interview‐Revised; ADOS, Autism Diagnostic Observation Schedule; CARS, Childhood Autism Rating Scale; MFI, mean fluorescence intensity; T_CM_, T central memory; T_EM_, T effector memory; T_EMRA_, T effector memory re‐expressing CD45RA; T_N_, T naïve.

Adaptive immune cells, primarily T and B cells, constituted approximately 70% of our immune‐targeted transcriptome profiles. Dysfunction of T lymphocytes may be linked to disturbances in behaviour and developmental functioning in ASD.^23^ We observed a significant reduction in the frequency, but not the absolute numbers, of CD3^+^ T cells in ASD individuals (Figure 2A). Using mFCM, we examined the frequencies and absolute numbers of naïve (T_N_), central memory (T_CM_), effector memory (T_EM1/2/3_) and terminal effector memory re‐expressing (T_EMRA_) phenotypes within CD4^+^ and CD8^+^ T cell subsets, identified by CD45RA, CD27 and CCR7 markers (Figure S3). Consistent with previous studies,^24^, ^25^ ASD individuals exhibited reduced frequencies of CD4^+^ T cells, albeit not in absolute numbers. We observed a significant decrease in CD4^+^ T_N_, T_CM_ and T_EM1_ populations in ASD, confirming both percentage and absolute counts. Conversely, populations of T_EM2/3_ and T_EMRA_ within the CD4^+^ T cell compartment were increased in ASD patients (Figure 2B). Regarding CD8^+^ T cells, no significant differences were observed in overall percentages or absolute counts between groups. However, trends paralleled those seen in CD4^+^ T cells, with notable reductions in CD8^+^ T_N_, T_CM_ and T_EM1_ populations, and increases in CD8^+^ T_EM2/3_ and T_EMRA_ populations (Figure 2C). No significant variations in activated CD4^+^ T or CD8^+^ T cells were observed between ASD and TD individuals (Figure S4A). Thus, peripheral T cells in ASD patients display a reduced proportion of naïve phenotypes and an increased proportion of terminal effector memory phenotypes, reflecting T cell activation and suggesting a state of ongoing chronic antigenic stimulation or inflammation in these patients. Further analysis of immune cell composition in bulk RNA‐seq data using the ImmuneCellAI^26^ database corroborated the transition from naïve to effector memory states in T cells (Figure 2D), aligning with the mFCM results. Additionally, the frequency of CD57^+^ senescent T cells was significantly elevated in ASD patients (Figure 2E), indicating lower proliferative capacity in periphery T cells.^27^, ^28^ As previously reported,^25^ the number of T regulatory (Treg) cells was significantly lower in ASD patients compared to TD individuals. We also discovered a noticeable reduction in naïve Tregs and an increase in memory Tregs (Figure 2F and Figure S4B). Analysis of T helper (Th) subsets indicated a shift from Th1 to Th2 subsets, whereas Th17 cells showed no difference between ASD and TD patients, suggesting a more active type 2 immune response in ASD (Figure 2G and Figure S4C). The populations of PD‐1^hi^CXCR5^+^ T follicular helper, PD‐1^hi^CXCR5^−^ T peripheral helper (Tph) and PD‐1^−^CXCR5^+^ pro‐Tph cells were similar between the two groups (Figure 2H and Figure S4D). Tph cell subsets, involved in extra‐follicular B cell activation,^29^ and their aberrant proliferation are commonly observed in autoimmune diseases and infections.^30^, ^31^ We characterized PD‐1^hi^CXCR5^−^ Tph cells within CD45RA^−^ memory CD4^+^ T cells and subsequently defined four Tph subsets (Tph1, Tph2, Tph17 and Tph1‐17) based on the cell surface expressions of CXCR3 and CCR6 (Figure S4E). The percentages of Tph1, Tph17 and Tph1‐17 cells were markedly decreased in ASD patients compared to TD individuals, whereas the proportion of Tph2 cells was increased (Figure 2I).

To further clarify T cell heterogeneity, we projected CD4^+^ T and CD8^+^ T cell scRNA‐seq data onto a 2D UMAP, identifying four subsets each (Figure 2J,K and Figure S4F), with the top ten DEGs for each subset presented in Figure S4G. Using signatures of naïve, Treg, exhaustion and cytotoxicity profiles, we computed transcriptional scores for both CD4^+^ and CD8^+^ subsets. CD4T_c01_LEF1 and CD8T_c01_CCR7 exhibited the strongest signature characteristic of naïve cells. CD4T_c02_FOS and CD8T_c03_ZNF683 showed higher exhaustion scores than other signatures, and CD4T_c03_ANXA2_FOXP3 demonstrated the most pronounced Treg signature (Figure 2L,M). Notably, CD8T_c02_NKG7 displayed significant cytotoxic activity and expressed higher levels of *KLRG1*, *GZMK*, *GZMH*, *GZMA* and *GZMB*, indicative of the CD8^+^ T_EM_ phenotype (Figure S4H). We subsequently determined the DEGs between ASD and TD subsets at single‐cell level (Figure S4I) and revealed an interferon‐stimulated gene (ISG) enrichment in ASD patients, a feature commonly seen in autoimmune diseases.^32^, ^33^ By performing ingenuity pathway analysis (IPA) using the IPA software, we discovered that genes associated with cell death and interferon signalling pathways were upregulated in ASD patients compared to TD individuals (Figure S5). These findings suggest that T cells in ASD may initiate cell death programmes while being activated, a phenomenon commonly seen in infectious diseases where immune cells are damaged by pathogens or antigens.^34^ Interestingly, these T cells expressed elevated levels of IFN‐γ and TNF‐α (Figure 2N, Figure S6). Certain T cell subpopulations correlated with disease severity. We observed that a higher abundance of CD4^+^ T_EM2_ cells correlates with the Autism Diagnostic Observation Schedule (ADOS) scores, suggesting a potential pathophysiological role in ASD. These cells also showed an inverse correlation with DQ/IQ, which negatively correlates with ASD severity (Figure 2O). Similarly, a decreased population of CD8^+^ T_EM1_ cells was inversely correlated with both Autism Diagnostic Interview‐Revised (ADI‐R) and ADOS scores (Figure 2P). These findings indicate that the presence of ongoing immune activation of T cells in patients with ASD is closely related to disease occurrence.

### 2.3 NK subsets displayed heightened activity but reduced cytotoxicity in ASD individual

**FIGURE 3:**
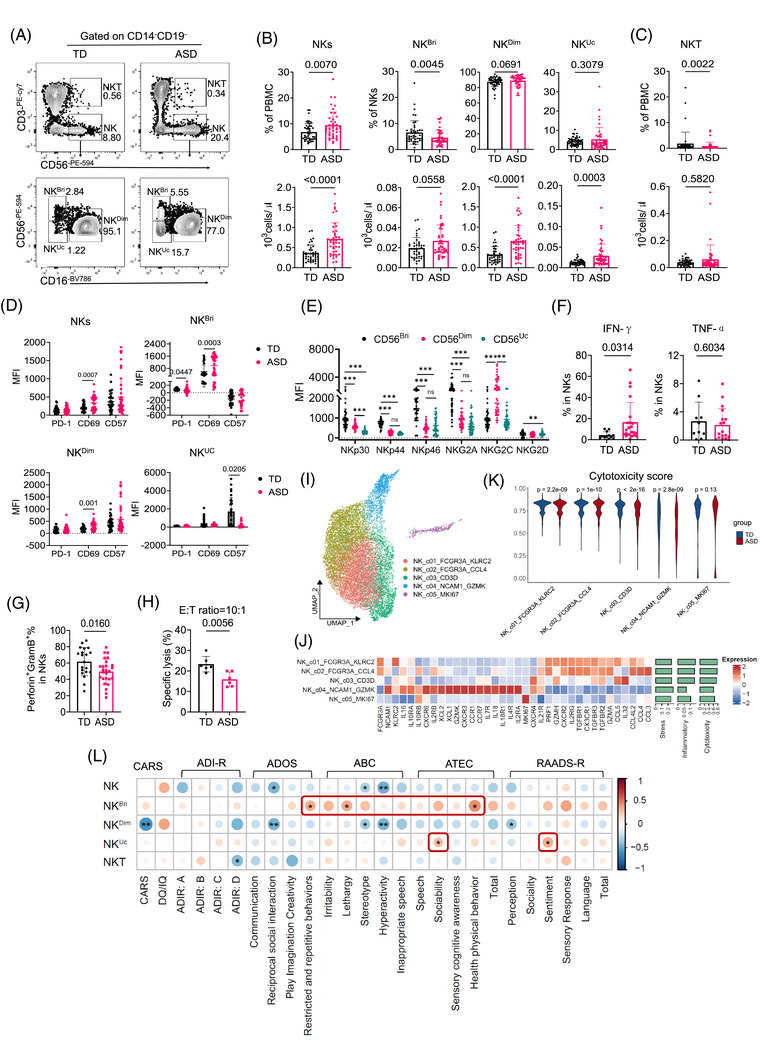
Natural killer (NK) cell heterogeneity in autism spectrum disorder (ASD) and its association with clinical metadata. (A and B) Representative flow cytometry gating plot (A) and proportions and absolute counts of NK cells and NK cell subsets (B) in ASD patients (*n* = 44) and typically developing (TD) individuals (*n* = 46). (C) Proportion and absolute count of NKT cells in ASD patients (*n* = 44) and TD individuals (*n* = 46). (D) Comparative surface expression levels of PD‐1, CD69 and CD57 on NK cell subsets from ASD patients (*n* = 28) and TD individuals (*n* = 23). (E) Comparative surface expression levels of activating receptors (NKp30, NKp44, NKp46, NKG2C and NKG2D) and inhibitory receptors (NKG2A) between NK cell subsets. (F) Percentage of IFN‐γ and TNF‐α positive NK cells in samples from ASD patients (*n* = 23) and TD individuals (*n* = 12). (G) Percentage of perforin and granzyme B positive NK cells in ASD patients (*n* = 26) and TD individuals (*n* = 20). (H) Comparison of NK cell cytotoxicity between ASD and TD samples (*n* = 6 per group) at an effector‐to‐target (E:T) ratio of 10:1. (I) Uniform manifold approximation and projection (UMAP) embedding of 13 632 NK cells from all single‐cell RNA sequencing (scRNA‐seq) samples. Each point represents one cell. Cells coloured according to the cell cluster. (J) Heatmap visualizing the row‐scaled average expression of functional genes in NK cells, with bar plots showing the AUCell index of signature gene sets. (K) Violin plots illustrating the AUCell index for NK functional gene sets among NK subsets. Red: ASD patients; blue, TD individuals. *p* values were calculated via the two‐tailed Wilcoxon test and adjusted by the Benjamini–Hochberg method. (L) Correlation matrix between the frequencies of NK cell subsets and clinical diagnostic scale scores. The circle size corresponds to the absolute value of the Spearman correlation coefficient, with red (blue) colour indicating a positive (negative) correlation. ^*^ *p *< .05, ^**^ *p* < .01. For B–H, data are presented as mean ± standard deviation (SD), and two‐tailed unpaired *t*‐tests were applied. NK^Dim^, conventional CD56^dim^CD16^pos^ NK cells; NK^Uc^, unconventional CD56^dim^CD16^neg^ NK cells; NK^Bri^, conventional CD56^bright^CD16^neg^ NK cells. ABC, autistic behaviour checklist; ADI‐R, Autism Diagnostic Interview‐Revised; ADOS, Autism Diagnostic Observation Schedule; ATEC, Autism Treatment Evaluation Checklist; CARS, Childhood Autism Rating Scale; MFI, mean fluorescence intensity; RAADS‐R, Ritvo Autism Asperger Diagnostic Scale‐Revised.

It has been reported that NK cells are elevated in peripheral blood of ASD patients and may play an essential role.^13^, ^14^ To investigate the phenotype of NK cells in ASD individuals, we assessed the frequency and absolute count of NK cells among CD14^−^CD19^−^ lymphocytes (Figure 3A). We found a significantly higher proportion and absolute count of NK cells in ASD patients compared to TD individuals (Figure 3B), aligning with prior reports.^14^ Specifically, there was a substantial expansion in absolute counts of conventional CD56^dim^CD16^pos^ NK cell (NK^Dim^) and unconventional CD56^dim^CD16^neg^ NK cell (NK^Uc^) subsets^35^ within NK cells, whereas the absolute count of conventional CD56^bri^CD16^neg^ NK cells (NK^Bri^) increased but showed no significant difference between the groups (Figure 3B). Additionally, the frequency of CD56^bri^ CD3^pos^ NK cells (NKT) was significantly reduced in ASD individuals, although their absolute counts remained unchanged (Figure 3C). We observed increased expression of activation markers CD69 and CD57 in NK subsets of ASD individuals, suggesting early activation of these subgroups (Figure 3D). To further delineate each NK subset, we analysed the expression of activating receptors (NKp30, NKp44, NKp46, NKG2C and NKG2D) and inhibitory receptors (NKG2A). NK^Uc^ cells exhibited characteristics similar to NK^Dim^, evidenced by their low expression of NKp30, NKp46, NKG2A and NKG2D (Figure 3E). We also measured the expression levels of inflammatory cytokines (IFN‐γ and TNF‐α) in unstimulated NK cells and detected a significant elevation in IFN‐γ expression in ASD group (Figure 3F), aligning with a previous study.^36^ Conversely, the production of perforin and granzyme B in NK cells from ASD individuals was reduced (Figure 3G). Further assessing NK cell cytotoxicity against K562 cells, we confirmed markedly impaired cytotoxicity in ASD patients (Figure 3H). Consistently, the LDH release assay also demonstrated significantly reduced NK cell cytotoxicity in ASD patients at 4 and 6 h after co‐culture with K562 cells, compared to TD controls (Figure S7A).

Our scRNA‐seq analysis identified 13 632 NK cells, which were subclustered into five subsets (Figure 3I). These subsets were termed on the basis of their transcriptional signatures (Figure S7B). NK_c01_FCGR3A_KLRC2 and NK_c02_FCGR3A_CCL4 exhibited the highest expression of *FCGR3A* and lower levels of *NCAM1* (CD16^+^ NK^Dim^), whereas NK_c04_NCAM1_GZMK showed the highest levels of *NCAM1* and lower levels of *FCGR3A* (CD16^−^ NK^Bri^). NK_c03_CD3D was characterized by upregulation of *CD3D* (NKT), and NK_c05_MKI67 upregulated *MKI67* (proliferating NK), displaying the lowest transcriptional scores of inflammatory and cytotoxicity signatures and the highest score of stress signature (Figure 3J and Figure S7C). The cytotoxicity signature scores of different NK cell subsets were significantly reduced in individuals with ASD compared to TD (Figure 3K), consistent with the mFCM data. We determined DEGs between NK cells from ASD and TD at the single‐cell level, revealing an enrichment of ISGs (Figure S7D), similar to T cells. Notably, genes associated with interferon signalling, cell death and antiviral response pathways were upregulated in ASD patients, as determined by IPA (Figure S8). Among NK cell subsets, increased NK^Bri^ and NK^Uc^ subsets showed associations with ASD scoring metrics such as ADOS and Autism Treatment Evaluation Checklist (ATEC) scores (Figure 3L). Our results suggest that the previously unreported NK^Uc^ population was rapidly expanded in ASD patients, accompanied by elevated proinflammatory capacity and impaired NK‐cell cytotoxicity, which correlates with the severity of ASD disease.

### 2.4 Myeloid subsets exhibited high‐inflammatory phenotypes correlating with ASD pathogenesis

**FIGURE 4:**
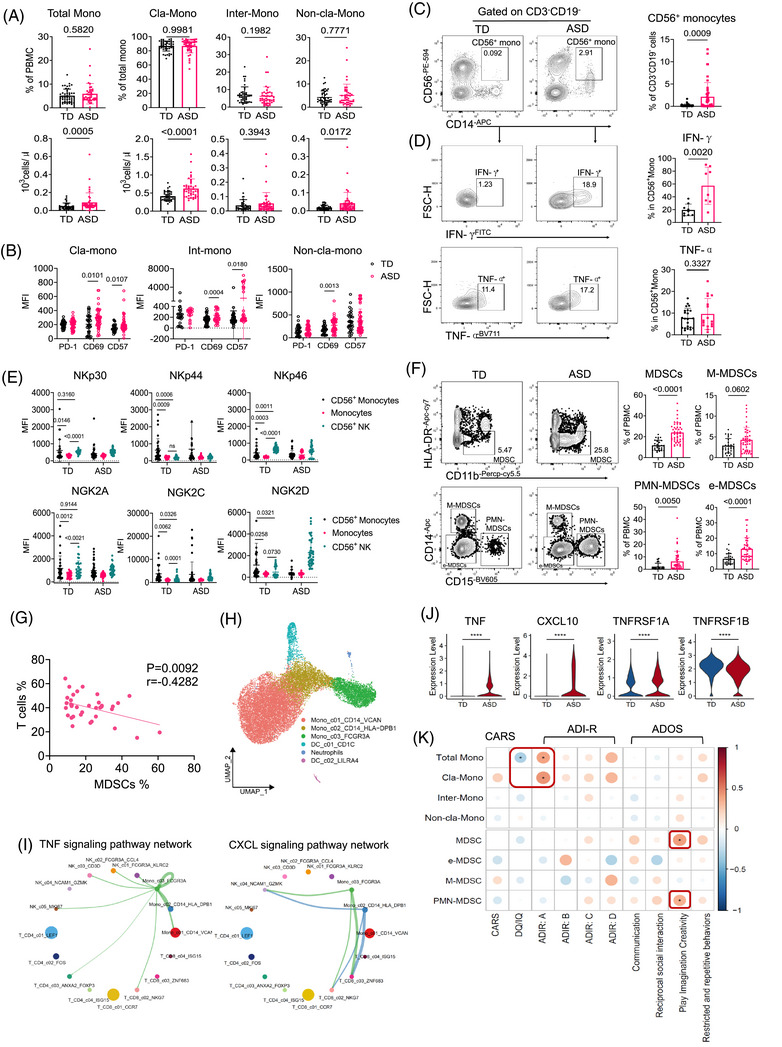
Myeloid cell heterogeneity in autism spectrum disorder (ASD) and its association with clinical metadata. (A) Proportions and absolute counts of monocytes and their subsets in ASD patients (*n* = 44) and typically developing (TD) individuals (*n* = 46). The gating strategy is detailed in Figure S3. (B) Comparative surface expression levels of PD‐1, CD69 and CD57 on monocyte subsets between ASD patients (*n* = 44) and TD individuals (*n* = 33). (C) Representative flow cytometry gating plot (left) and the percentage of CD56‐positive monocytes in CD3^−^CD19^−^ peripheral blood mononuclear cells (PBMCs) (right) isolated from ASD patients (*n* = 46) and TD individuals (*n* = 33). (D) Representative flow cytometry gating plot and the percentage of IFN‐γ, TNF‐α positive cells in CD56^+^ monocytes isolated from ASD patients (*n* = 8–16) and TD individuals (*n* = 9–22). (E) Surface expression levels of activating receptors (NKp30, NKp44, NKp46, NKG2C and NKG2D) and inhibitory receptors (NKG2A) on monocyte subsets compared between ASD patients (*n* = 37) and TD individuals (*n* = 28). (F) Representative flow cytometry gating plot and proportions of MDSC subsets in ASD patients (*n* = 44) and TD individuals (*n* = 46). (G) Correlation of T cell and MDSC frequencies in ASD patients (*n* = 36). (H) Uniform manifold approximation and projection (UMAP) embedding of 18 808 monocytes from all single‐cell RNA sequencing (scRNA‐seq) samples. Each point represents one cell. Cells coloured according to the cell cluster. (I) Inferred TNF and CXCL signalling pathway networks in ASD scRNA‐seq profile. Different colours in circles represent various cell types, with circle sizes proportional to the number of cells in each subset. The width of connecting lines represents the communication probability. (J) Violin plots showing expression of the TNF ligand, the TNFRSF1A and TNFRSF1B receptors, the CXCL10 ligand and the CXCR3 receptor between ASD (red) and TD (blue) groups in Mono_c03_FCGR3A subset. P values were calculated via the two‐tailed Wilcoxon test and adjusted by the Benjamini–Hochberg method. ^****^ *p* < .0001. (K) Correlation matrix between the frequencies of monocyte subsets and clinical diagnostic scale scores. The circle size corresponds to the absolute value of the Spearman correlation coefficient, with red (blue) colour indicating a positive (negative) correlation. ^*^ *p* < .05. For **A–F**, data are presented as mean ± standard deviation (SD), and two‐tailed unpaired *t*‐tests were applied. ADI‐R, Autism Diagnostic Interview‐Revised; ADOS, Autism Diagnostic Observation Schedule; CARS, Childhood Autism Rating Scale; Cla‐Mono, classical‐monocytes; e‐MDSCs, early‐MDSCs; Int‐Mono, Intermediate‐monocytes; MDSCs, myeloid‐derived suppressor cells; MFI, mean fluorescence intensity; M‐MDSCs, monocytic‐MDSCs; Non‐cla‐Mono, non‐classical‐monocytes; PMN‐MDSCs, polymorphonuclear‐MDSCs.

Previous research studies have noted increased monocyte numbers in ASD patients^7^, ^37^; which we confirmed through significantly higher absolute counts of monocyte subsets in ASD compared to TD individuals (Figure 4A). These expanded monocytes demonstrated increased expression of activation markers CD69 and CD57 in ASD individuals (Figure 4B). Notably, CD56^+^ monocytes, co‐expressing CD14 and CD56, exhibited direct cytolytic activity towards dysfunctional cells^38^ and were increased in ASD but present in very low frequencies in TD individuals (Figure 4C). These CD56^+^ monocytes produced more IFN‐γ and TNF‐α than those derived from TD individuals, suggesting they may represent a new source of inflammatory factors (Figure 4D). Further analysis revealed that CD56^+^ monocytes exhibited the highest expression levels of natural cytotoxicity receptors and NK receptor complexes compared to both monocytes and NK cells, suggesting superior cytotoxic activity and cytokine production capabilities (Figure 4E). We also reported, for the first time, a significant increase in myeloid‐derived suppressor cells (MDSCs) and their subsets (except for monocytic‐MDSCs) in ASD patients compared to TD individuals, identified by CD11b, HLA‐DR, CD14 and CD15 markers (Figure 4F). Our data further demonstrated a significant negative correlation between MDSCs and T cells (Figure 4G). This finding suggests that the activated T cells in ASD may be suppressed by MDSCs,^39^ underscoring their potential role in modulating immune responses in ASD.

We projected myeloid cell scRNA‐seq data onto a 2D UMAP, identifying three monocyte subsets, two DC subsets and a neutrophil subset based on transcriptional signatures (Figure 4H and Figure S9A). Mono_c01_CD14_VCAN displayed the highest *CD14* expression (classical‐monocytes), Mono_c03_FCGR3A showed the highest *FCGR3A* expression (non‐classical‐monocytes), Mono_c02_CD14‐HLA‐DPB1, with moderate *CD14* and *FCGR3A* expression, displayed the highest transcriptional score for the MDSC signature (Figure S9B,C). DCs were classified into conventional DCs (DC_c01_CD1c) and plasmacytoid DCs (DC_c02_LILRA4). All three monocyte subsets were expanded in ASD individuals (Figure S9D), consistent with the mFCM data. To investigate alterations in cell–cell communication between adaptive and innate immune subsets in ASD, we systematically analysed potential cell–cell interactions by assessing the co‐expression of established ligand–receptor pairs across T cells, NK cells and monocytes. We found that interactions involving the TNF and CXCL signalling pathways were activated only in ASD monocytes and T/NKs (Figure 4I and Figure S9E), but not in TD group. Mono_c03_FCGR3A exhibited the highest interaction score with adaptive immune subsets, particularly through ligand–receptor pairs *TNF*‐*TNFRSF1B*, *TNF*‐*TNFRSF1A* and *CXCL10*‐*CXCR3* in ASD individuals (Figure S9E). Additionally, Mono_c03_FCGR3A expressed elevated levels of *TNF*, *CXCL10*, *TNFRSF1A* and *TNFRSF1B* in ASD compared to TD (Figure 4J). TNF and CXCL10 are critical cytokines involved in inflammatory responses and immune cell activation, associated with various autoimmune diseases.^40^, ^41^ Further, DEG analysis revealed an enrichment of ISGs in ASD (Figure S9F). IPA showed upregulation of genes related to cell death and interferon signalling pathways in ASD monocyte subsets (Figure S10). Clinically, we observed that a higher abundance of monocytes correlated with ASD occurrence indicators (ADI‐R score) and inversely with DQ/IQ. Notably, an increased MDSC population correlated with ADOS scores (Figure 4K). These data suggest that myeloid cells, particularly activated inflammatory monocytes and MDSCs, play a potential pathophysiological role in ASD.

### 2.5 Characterization of the plasma inflammatory factors differentially presented in ASD patients

**FIGURE 5:**
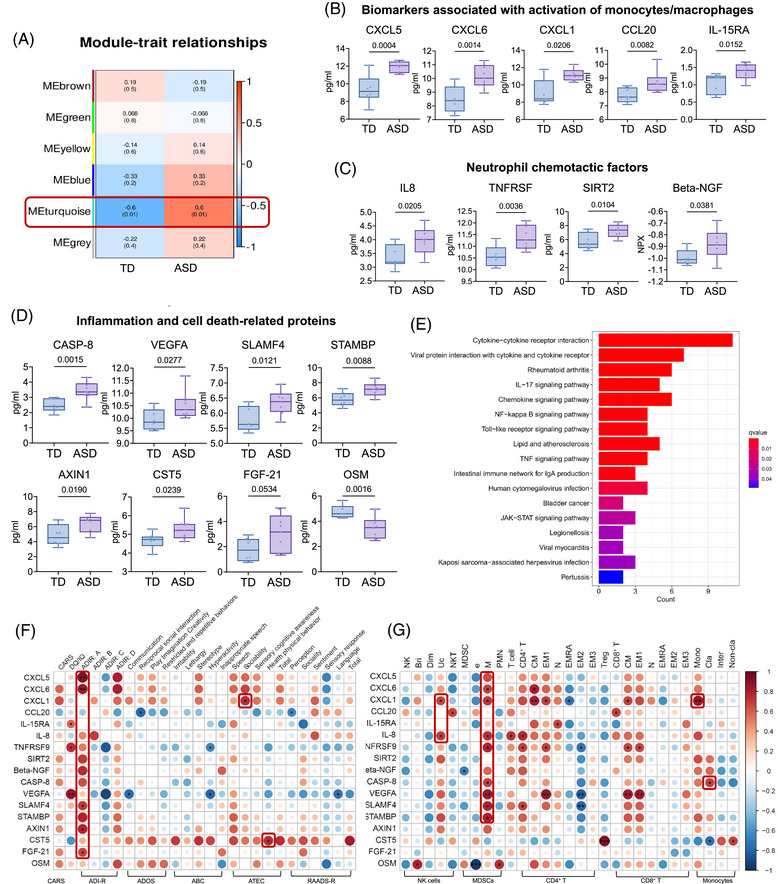
Characterization of differentially expressed plasma immune and inflammatory factors in autism spectrum disorder (ASD) patients. (A) Module–trait relationships in two groups, displayed in heatmaps where red indicates a positive correlation and blue indicates a negative correlation. Correlation *p* values are shown in brackets. A red box highlights the turquoise module. (B–D) Box plots illustrating plasma protein concentrations associated with monocytes/macrophages (B), neutrophil chemotactic factor (C) and inflammation and cell death‐related proteins (D) in ASD patients (*n* = 9) and typically developing (TD) individuals (*n* = 8). Data are presented as mean ± standard deviation (SD), and two‐tailed unpaired *t*‐tests were applied. (E) Bar plot showing the top enriched Kyoto Encyclopedia of Genes and Genomes (KEGG) pathways of significantly upregulated plasma proteins from ASD patients. (F and G) Correlation matrix between plasma proteins and clinical diagnostic scale scores (F) or immune cell populations (G). The circle size corresponds to the absolute value of the Spearman (F) and Pearson (G) correlation coefficients, with red (blue) colour indicating a positive (negative) correlation. ^*^ *p* < .05; ^**^ *p* < .01; ^***^ *p* < .001. ABC, autistic behaviour checklist; ADI‐R, Autism Diagnostic Interview‐Revised; ADOS, Autism Diagnostic Observation Schedule; ATEC, Autism Treatment Evaluation Checklist; CARS, Childhood Autism Rating Scale; RAADS‐R, Ritvo Autism Asperger Diagnostic Scale‐Revised.

We conducted comprehensive profiling of 92 plasma proteins in ASD patients using the Olink platform, revealing significant alterations in various inflammatory factors. Weighted co‐expression network analysis (WGCNA) identified modules of correlated proteins, with the turquoise module significantly associated with ASD status (Figure 5A). Several proteins with altered concentrations in ASD were identified, including activation markers of monocytes/macrophages (Figure 5B), neutrophil chemotactic factor (Figure 5C) and inflammation‐related proteins (Figure 5D). These results corroborate the increased activation of ASD monocytes suggested by mFCM data and transcriptional changes observed in scRNA‐seq data. Pathway analysis highlighted immune abnormalities associated with the plasma protein profile, particularly involving the chemokine signalling pathway and cytokine–cytokine receptor interaction (Figure 5E). We investigated proteins associated with disease severity and found significant correlations for several, including CXCL5, CXCL6, SLAMF4, FGF‐21 and AXIN1 with the ADI‐R score. Additionally, CST5 and CXCL1 were positively correlated with disease severity measured by the ATEC score (Figure 5F). Further analysis revealed associations between proteins and immune cell populations, with agglomerated NK^Uc^ correlating with CXCL1 and IL8, agglomerated NK^Bri^ with OSM and expanded M‐MDSCs with CXCL5, CXCL6, SLAMF4 and other proteins. Moreover, total monocyte frequencies correlated with CXCL1 (Figure 5G). These findings indicate that proteins involved in recruiting inflammatory myeloid cells, particularly monocytes and MDSCs, strongly correlate with ASD severity.

### 2.6 Plasma metabolic profiling from ASD individuals reveals disease‐specific perturbations

**FIGURE 6:**
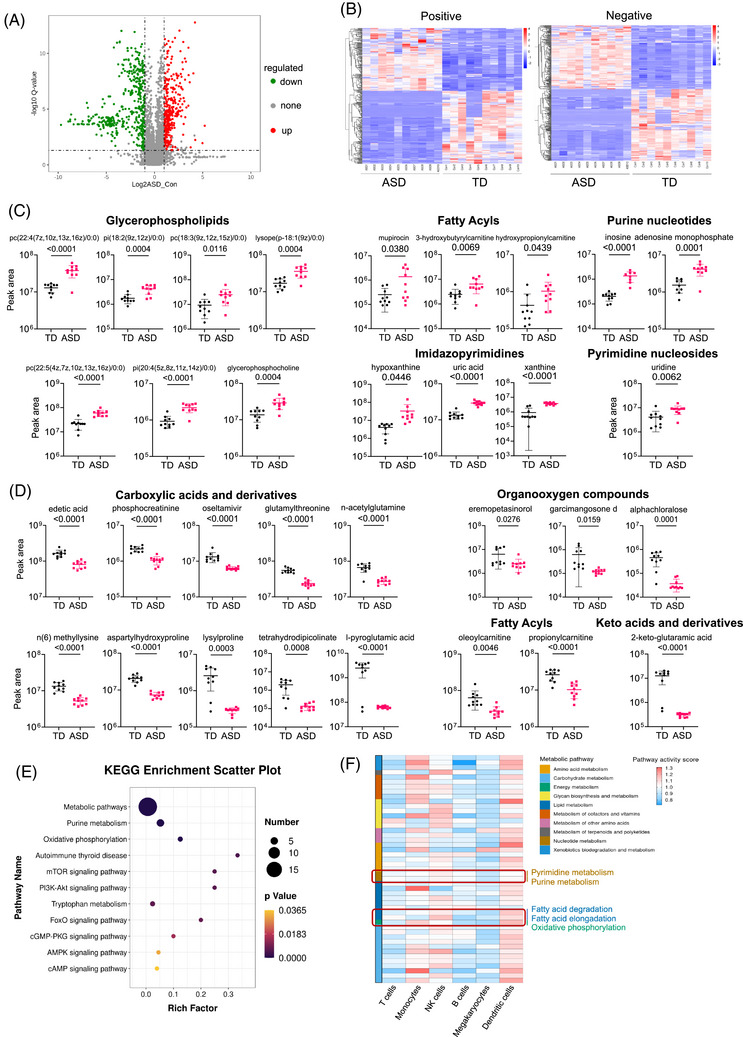
Plasma metabolic profiling from autism spectrum disorder (ASD) individuals reveals disease‐specific perturbations. (A) Volcano plot displaying the differentially quantitative metabolites between ASD and typically developing (TD) groups. Each red dot represents a metabolite significantly altered with an adjusted *Q* value < .05 and fold change ≥ 2, analysed using a two‐tailed Wilcoxon test. (B) Heatmap illustrating the differential metabolites between the ASD and TD groups, acquired in positive ion mode (right) and negative ion mode (left). (C and D) Abundance of differential metabolites selected from (B), which were upregulated (C) or downregulated (D) in ASD group. Data are presented as mean ± SD, and two‐tailed unpaired *t*‐tests were applied. (E) Scatter plot highlighting the top enriched Kyoto Encyclopedia of Genes and Genomes (KEGG) pathways of significantly upregulated differential metabolites in ASD patients. (F) Heatmap visualizing the metabolic pathway activity scores of six major cell lineages in single‐cell RNA sequencing (scRNA‐seq) profiles. The red box indicated the nucleotide metabolism, energy metabolism and lipid metabolism pathways were highlighted.

Given the close association between immune responses and metabolic changes, we further identified and quantified plasma metabolites in ASD patients and TD individuals. Quantitative analysis revealed significant differences (Figure S11A), with 763 distinct metabolites altered in ASD individuals (Figure 6A). These differential metabolites were grouped into four clusters based on change trends and ion modes (Figure 6B and Figure S11B), with representative metabolites shown in Figure 6C,D. Notably, glycerophospholipids, purine nucleotides, imidazopyrimidines and pyrimidine nucleosides were enriched in ASD individuals (Figure 6C), whereas carboxylic acids, organooxygen compounds and keto acids and derivatives were decreased (Figure 6D). Some fatty acyls were elevated, whereas others decreased in ASD group. Kyoto Encyclopedia of Genes and Genomes (KEGG) pathway analysis indicated that metabolic changes in ASD predominantly involved purine metabolism and oxidation phosphorylation, with some metabolites enhancing immune signalling pathways like mTOR and PI3K‐Akt signalling pathways (Figure 6E). To further investigate metabolic pathway activity across various immune compositions, we utilized scRNA‐seq profiles to perform metabolic pathway activity scoring. Metabolites associated with nucleotide metabolism, such as purine nucleotides and pyrimidine nucleosides, showed increased activity in NK cells, whereas those involved in energy metabolism, specifically oxidative phosphorylation, were more active in myeloid cells. Similarly, lipid metabolism metabolites, particularly those related to fatty acid degradation, also exhibited elevated activity in NK cells (Figure 6F and Figure S11C). Correspondingly, mFCM analysis confirmed an increase in the frequency and absolute numbers of monocyte and NK cell subsets in ASD, correlating positively with disease severity. We also identified metabolites significantly associated with ASD severity, including glycerophospholipids, carboxylic acids and their derivatives and fatty acyls, which correlated with the ADI‐R score (Figure S11D,E). These metabolic alterations, closely tied to shifts in immune cell composition, may play a pivotal role in the pathogenesis and progression of ASD.

### 2.7 Alteration of peripheral immune composition in ASD individuals following risperidone administration

**FIGURE 7:**
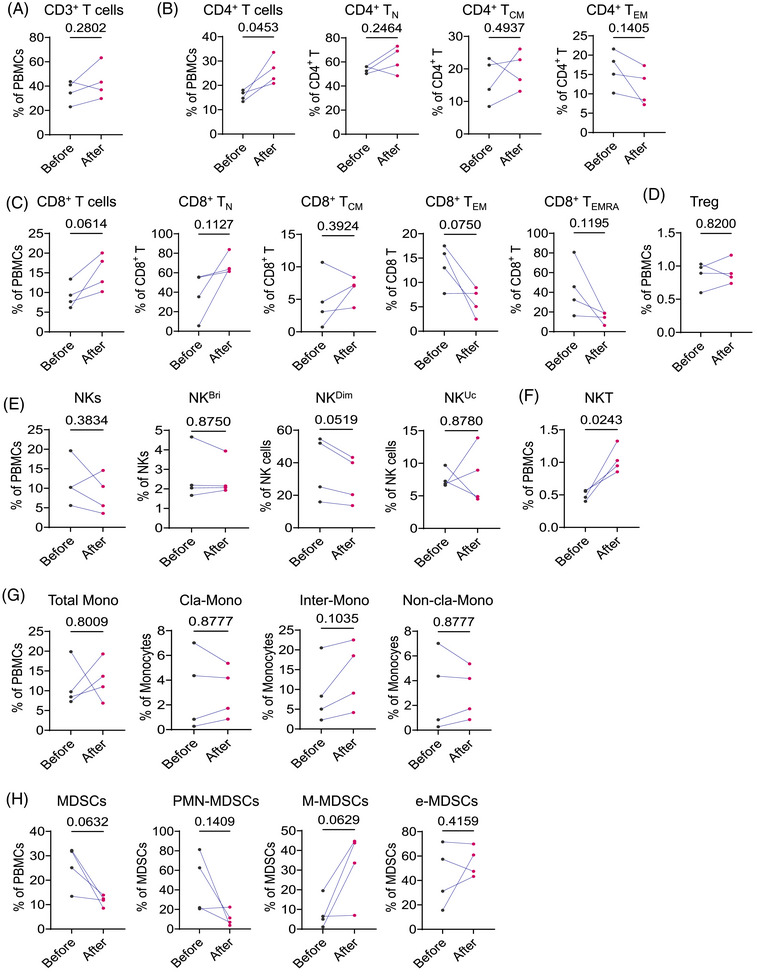
Alteration of peripheral immune composition in autism spectrum disorder (ASD) individuals following risperidone administration. Proportions of CD3^+^ T cells (A), CD4^+^ T cell subsets (B), CD8^+^ T cell subsets (C), T regulatory (Tregs) (D), natural killer (NK) cell subsets (E and F), monocyte subsets (G) and myeloid‐derived suppressor cells (MDSC) subsets (H) from ASD patients before and after risperidone treatment (*n* = 4). Two‐tailed paired *t*‐tests were applied.

Risperidone has been shown to effectively reduce the severity of ASD symptoms^42^ and lower peripheral inflammatory mediators in ASD.^43^ Additionally, it inhibits the generation of inflammatory cytokines like IL‐1β and TNF‐α in mild neuroinflammation models, demonstrating its anti‐inflammatory properties.^44^ In our study, we recruited four ASD patients undergoing risperidone treatment at doses between .33 and .5 mg, administered once daily at bedtime. Peripheral blood samples were collected from these patients before and after 8 weeks of risperidone treatment to evaluate changes in immune cell subsets associated with improvements in ASD symptoms. Following risperidone treatment, there was an expansion in the percentage of CD3^+^ T cells, notably CD4^+^ T cells, and a trend towards elevation in CD8^+^ T cells and Tregs (Figure 7A–D). Mature NK cells showed a decreasing trend (Figure 7E), whereas NKT cells significantly increased (Figure 7F). Changes in monocytes were less pronounced (Figure 7G), whereas subsets of MDSC and polymorphonuclear‐MDSCs exhibited a decline (Figure 7H). Following risperidone treatment, changes in immune cell composition were observed, further supporting the potential role of the immune system in ASD.

## 3 DISCUSSION

Immunological dysfunction plays a crucial role in neurodevelopmental deficits in ASD. A comprehensive understanding of immune responses is essential for evaluating treatment efficacy, predicting disease outcomes and elucidating disease pathogenesis. By integrating clinical observations, alternations in immune composition and plasma analytics, we have developed a comprehensive and integrated perspective on ASD.

T cell dysfunction has been implicated in various neurodevelopmental^45^ and neurodegenerative disorders.^46^ Our findings of a notable reduction in T cell frequency, characterized by a shift from naïve to effector memory states, align with previous research^23^ and suggest an accelerated ageing process and chronic activation state. This observation is consistent with chronic infections and autoimmune diseases,^47^ conditions frequently associated with ASD. Furthermore, the reported association between prenatal maternal infections^48^ or autoimmune diseases^49^ and ASD aetiology underscores the relevance of these findings. Maternal immune activation^50^ and prenatal toxins exposure^51^ have been shown to skew the immune balance towards Th2 dominance, potentially impacting foetal brain development. Our observed shift towards Th2 dominance, indicated by altered Th1/Th2 ratios and elevated Th2 responses, correlates with increased susceptibility to allergies, commonly reported in ASD individuals.^52^ Elevated B cell count (Figures S12 and S13) and pro‐inflammatory cytokines^53^ further support the significance of Th2 responses in ASD pathology.

Within the effector memory compartment, we observed expansions of CD4⁺ T_EM2_, T_EM3_ and T_EMRA_ subsets in ASD. Notably, only the T_EM2_ subset demonstrated a significant positive correlation with ADOS scores, suggesting a closer link with clinical severity. TEM2 cells, defined as CD27^−^CD45RA^−^CCR7⁺, retain lymphoid‐homing capacity through CCR7, which directs T cell migration to secondary lymphoid organs through CCL19/CCL21 gradients. These CCR7⁺ memory T cells can recirculate between blood and lymphoid organs, supporting continuous immune surveillance.^54^, ^55^ The combination of CCR7 expression and CD27 loss indicates an advanced differentiation state with maintained lymphoid trafficking capacity,^56^ which may contribute to their association with ASD clinical severity. In contrast, T_EM3_ (CD27^−^CD45RA^−^CCR7^−^) and T_EMRA_ (CD27^−^CD45RA⁺CCR7^−^) subsets, although also increased, did not correlate with severity scores, suggesting they may reflect broader features of chronic immune activation and terminal differentiation that are not directly proportional to symptom severity. These findings highlight T_EM2_ cells as a potential severity‐associated immune signature in ASD and underscore the heterogeneity within the expanded effector memory pool.

NK cells in ASD patients exhibit increased activity but reduced cytotoxicity, a phenomenon consistent with chronic inflammation and overactive immune responses. Despite the expansion of the NK^Dim^ subset, cytotoxic function is compromised, as evidenced by the decreased production of perforin and granzyme B. The significant expansion of the atypical NK^Uc^, previously associated with various clinical conditions including malignancies and infectious diseases,^57^, ^58^, ^59^ suggests a role in ASD pathology. The potential origin of NK^Uc^ cells, whether as precursors to NK^Dim^ cells or differentiated NK cells, warrants further investigation.^60^ High levels of proinflammatory cytokines may drive NK^Uc^ expansion, and understanding this mechanism is crucial for elucidating the role of NK^Uc^ cells in ASD. In terms of clinical significance, we found that the NK^Bri^ subset, a major cytokine‐releasing population, and the expansion of the NK^Uc^ subset are significantly associated with the clinical severity of ASD.

We observed a marked expansion of myeloid populations, particularly CD56^+^ monocytes and MDSC subsets, in ASD. MDSCs, a heterogeneous population of immune cells known for their dual role in neuroinflammatory processes, can both exacerbate and mitigate neuroinflammation.^61^ On the one hand, MDSCs can secrete pro‐inflammatory cytokines and reactive oxygen species, thereby exacerbating neuroinflammation and contributing to neuronal damage.^62^ On the other hand, MDSCs may also exert immunosuppressive effects, attenuating excessive immune responses and mitigating neuroinflammatory damage.^63^ The elevation of MDSC subsets and their role in suppressing T cell activity suggest that these cells may emerge from the chronic inflammatory environment in ASD, modulating neuroinflammation by suppressing adaptive immune functions. The CD56^+^ monocyte population, characterized by heightened cytotoxicity and cytokine production, has also been associated with the development of autoimmune diseases, potentially contributing to the atypical immune responses seen in ASD.^64^ Accompanied by heightened cytokine levels^9^ and the upregulation of key immune regulatory genes,^10^ our scRNA‐seq data further confirmed the activation of pivotal signalling pathways between monocytes and other immune subsets, such as TNF and CXCL10, which are also elevated in both the cerebrospinal fluid and peripheral blood of ASD patients.^65^, ^66^ The activation of monocytes and upregulation of key immune regulatory genes further emphasize their underlying role in ASD pathology.

Our findings highlight the widespread activation of peripheral immune cells in ASD. There is upregulation of ISG‐related genes and activation of interferon and antiviral pathways across different immune subsets. These changes suggest a potential link between ASD pathogenesis and viral infections,^67^, ^68^ contributing to neuroinflammatory processes. The observed immune exhaustion, reflected by alterations in T cell phenotypes, monocyte functionality and NK cell cytotoxicity, indicates chronic antigen stimulation and inflammation.^69^

Some alterations in peripheral immune cell composition in ASD patients were observed after risperidone treatment, also suggesting a potential involvement of immune abnormalities in ASD pathogenesis and progression, though causality remains to be established. However, several immune subsets most strongly linked to disease severity in our cross‐sectional comparison with TD individuals showed little change after treatment. This may be due to two main reasons. First, risperidone primarily acts on neurotransmitter systems to reduce associated behavioural symptoms and does not directly target immune pathways. Stable immune subsets may reflect long‐term or trait‐like features of ASD, which are less likely to change over short treatment periods. Second, as noted in previous preclinical studies, risperidone can attenuate pro‐inflammatory cytokine production and modulate immune responses through distinct pathways depending on the physiological or pathological context.^70^, ^71^, ^72^ Clinically, it has been shown to elevate peripheral CD4⁺ and CD8⁺ T cell levels while reducing Th2 cells in first‐episode psychosis patients after 4 weeks of treatment^73^ and to reduce circulating pro‐inflammatory factors like eotaxin and MCP‐1 in ASD patients.^43^ Nevertheless, to our knowledge, no prior studies have specifically examined alterations in peripheral immune cells among individuals with ASD following risperidone administration. Our treatment analysis was exploratory, with a small sample size and short follow‐up, which inevitably limited statistical power and the ability to detect changes, especially in subsets linked to chronic immune remodelling such as T_EM_ cells and MDSCs.^74^, ^75^ These findings highlight the need for larger, randomized controlled trials and longitudinal studies to clarify the responsiveness of these severity‐associated immune subsets to risperidone and to evaluate their potential prognostic value.

The correlation between plasma biochemical changes and disease severity supports the role of immune component abnormalities in ASD. Our metabolomic analysis revealed the pivotal role of metabolites involved in purine metabolism and oxidative phosphorylation in ASD. Notably, Needham et al.^15^ identified dysregulated plasma and faecal metabolites linked to oxidative stress and mitochondrial impairment, subsequently supporting the link between metabolic disturbances and ASD pathogenesis. Our proteomic data demonstrated significant increases in biomarkers associated with monocyte/macrophage activation, neutrophil chemotactic factors and proteins related to inflammation and cell death in ASD, correlating with alterations in immune cell compositions and consistent with previous reports.^76^ These biomarkers, likely secreted by activated NK cells and monocytes, correlated with disease severity scores provide potential targets for diagnosis and therapeutic intervention.

Immune abnormalities may contribute to neurodevelopmental deficits in ASD by activating microglia, the central nervous system (CNS) resident macrophages essential for neuronal survival, apoptosis and synaptic remodelling.^77^ Dysregulated or excessive activation of microglia has been associated with an elevated risk of neurodevelopmental disorders.^78^ Peripheral inflammatory mediators can trigger microglial activation, disrupting synaptic pruning and circuit maturation. Activated microglial cells secrete pro‐inflammatory cytokines, including IL‐1β, IFN‐γ and TNF‐α, that alter synaptic plasticity and connectivity, linking peripheral immune activation to central neuroinflammation and circuit remodelling.^79^ Furthermore, dysregulated purine metabolism and mitochondrial/oxidative stress abnormalities, frequently reported in ASD, can enhance microglial reactivity and synaptic imbalance.^16^, ^80^ ISGs, which are activated by interferon signalling, have been shown to upregulate in brain endothelial cells, increasing blood–brain barrier (BBB) permeability^81^ and may also engage microglial IFN pathways, amplifying complement‐mediated synapse elimination.^82^ In addition, oxidative stress and other peripheral immune activation can also impair BBB integrity,^81^, ^83^ enabling systemic inflammatory signals to access the CNS. Together, these mechanisms provide a framework connecting peripheral immune dysregulation to the neurodevelopmental abnormalities in ASD.

In summary, our research underscores the importance of understanding the integrated immune network in ASD. By focusing on how various immune cell compositions alter and correlate with disease severity, we provide a framework for identifying new diagnostic and therapeutic targets. These insights pave the way for transitioning ASD treatment from symptom management to addressing the underlying immune dysregulation, offering a scientific basis for improved prevention and treatment strategies. Ultimately, these findings have the potential to guide the development of more personalized and effective therapeutic approaches, advancing both clinical management and research into the immunological mechanisms of ASD.

### 3.1 Limitations and future directions

Despite the comprehensive scope of our study, several limitations warrant acknowledgement. First, although our cohort exceeds many previous studies, the sample sizes for transcriptomic, proteomic and metabolomic analyses remain modest, potentially limiting statistical power and generalizability. Second, the cross‐sectional design cannot assess causality or temporal immune dynamics. Third, sample size constraints prevented stratification by ASD subtypes or comorbidities. Finally, the identified immune alterations require functional validation in vivo or in vitro to elucidate their mechanistic roles. Future studies should validate these findings in larger independent cohorts, incorporate longitudinal sampling to track immune changes and functionally characterize dysregulated immune subsets to advance biomarker discovery and immunomodulatory strategies for ASD.

## 4 METHODS

### 4.1 Subject details

The study enrolled a total of 63 ASD patients and 64 age‐ and sex‐matched TD controls. For mFCM analysis, samples were obtained from 44 ASD individuals (39 males and 5 females, aged 19–70 months) and 46 TD individuals (34 males and 12 females, aged 20–70 months). For RNA‐seq, 14 ASD samples (11 males and 3 females; 22–62 months) and 13 TD samples (7 males and 6 females, aged 28–56 months) were analysed. For scRNA‐seq, samples were collected from 5 ASD (3 males and 2 females, aged 24–48 months) and 5 TD (3 males and 2 females, aged 26–56 months) individuals. Samples used for proteomic (9 ASD patients and 8 TD controls) and metabolomic (10 ASD patients and 10 TD controls) analyses were partially overlapping with those used for mFCM. All participants were recruited from Guangzhou Women and Children Medical Center, Guangzhou Medical University, between June 2021 and December 2022. ASD diagnosis was confirmed by trained psychiatrists using the Childhood Autism Rating Scale, ADOS and ADI‐R. Additional assessments, including the Autistic Behaviour Checklist, ATEC and Ritvo Autism Asperger Diagnostic Scale‐Revised, aided in evaluating disease severity. Patients with comorbid epilepsy, attention deficit hyperactivity disorder or intellectual disability disorder were excluded. Sex‐ and age‐matched TD children undergoing routine medical examinations were recruited as controls. Exclusion criteria for the TD group included any neurological or psychiatric disorders, a family history of ASD or a recent history of infection. In both ASD and TD groups, individuals with a clinical diagnosis of autoimmune diseases—such as Henoch–Schönlein purpura, systemic lupus erythematosus or Kawasaki disease—were excluded. Informed consent was secured from all participants or their guardians. We collected blood routine test results, DQ/IQ scores and clinical diagnostic scale scores of ASD individuals. Information on age, sex and clinical diagnostic scale scores is listed in Tables S1 and S2. Spearman correlation coefficients were calculated using R package Hmisc (v4.2.7) to assess the associations between ASD clinical characteristics, and correlation significance was reported as *p* values calculated by function cor in R package stats (v4.2.2).

### 4.2 Plasma and PBMC isolation

Blood samples were collected into BD Vacutainer (EDTA) tubes. Plasma fractions were obtained by centrifugation at 2500 rpm at 4°C for 15 min, aliquoted and stored until use at −80°C until analysis. The remaining blood was diluted with phosphate buffered saline (PBS) to twice its original volume and layered over 3 mL of Ficoll per 2 mL of diluted blood in 15 mL tubes. Centrifuging at 600 × *g* for 30 min at room temperature (RT) allowed for the isolation of the PBMC layer, which was then transferred to a fresh conical tube and diluted again with PBS to twice its volume. After further centrifugation at 500 × *g* for 5 min at RT, the supernatant was discarded, leaving the PBMC pellet at the base of the tube. PBMC pellets were gently resuspended in 5 mL of red blood cell lysis buffer and incubated for 5 min at RT. The cells were washed twice with 10 mL PBS and centrifuged at 500 × *g* for 5 min at RT. Subsequently, PBMCs were resuspended in 2 mL PBS, and a 5 µL aliquot was diluted 1:10 (v/v) for cell counting. Cells were counted on a haemocytometer, and the cell concentration was adjusted to 1 × 10^6^ cells per mL. We then divided it into two fractions that were directly used for flow cytometry analysis and scRNA‐seq analysis.

### 4.3 Flow cytometry analysis

For cell surface marker staining, PBMCs were stained at a concentration of 1 × 10^6^ cells/mL using antibody cocktails (including CD3, CD4, CD8, CD19, CD127, CD25, CD45RA, CD27, CCR7, CD16, CD14, CD56, CD11b, CD11c, HLA‐DR, CD38, CD15, CD57, CD69, PD‐1, NKp30, NKp44, NKp46, NKG2C, NKG2D, NKG2A, CXCR5, CXCR3 and CCR6) for 30 min at 4°C. Cells were then washed twice with FACS buffer. For intracellular cytokine staining, cells were stimulated with a cell stimulation cocktail plus protein transport inhibitors (eBioscience) for 5 h. Subsequently, cells were fixed and permeabilized using Cytofix/Cytoperm buffer, followed by staining with antibodies against IL‐17a, IFN‐γ, TNF‐α, perforin and GZMB for 45 min at 4°C. After two additional washes, the cells were suspended in 200 µL FACS buffer. Stained cells were analysed using a BD LSRFortessa cell analyser. Antibody details are displayed in Table S6, and the gating strategy is available in Figure S3. The data were analysed with FlowJo software, and dimensional reduction analysis was performed using *t*‐SNE. Absolute cell counts are reported as cells/µL of blood collected from participants.

### 4.4 Single‐cell RNA sequencing

Sorted PBMCs were resuspended in PBS containing .04% bovine serum albumin, loaded into Chromium microfluidic chips with 5′ chemistry and barcoded with a 10× Chromium Controller (10× Genomics). RNA extracted from the barcoded cells was subsequently reverse transcribed, and sequencing libraries were constructed using the Chromium Single Cell 5′ v2 Reagent Kit (10× Genomics), following the manufacturer's instructions. Sequencing was conducted on an Illumina NovaSeq 6000, also following the supplier's instructions (Illumina).

### 4.5 Processing and analysis of scRNA‐seq data

Cell Ranger (v5.1.0) was used to align (GRCh38, Ensembl 98), filter, count barcodes and count unique molecular identifiers of sequencing reads from FASTQ files. Raw feature‐barcode matrixes were processed using the Seurat package (v4.3.0) in R environment^84^, ^85^; low‐quality cells, either disrupted or potential doublets, were excluded based on gene number (less than 200 or more than 5000) or mitochondrial content (more than 10%, Figure S2A). We used package DoubletFinder (v2.0.3)^86^ to identify and remove doublets. Filtered sample matrices were combined into a single object comprising 111 232 cells, with reads mapped to 22 411 human genes. This merged object was subsequently normalized with the LogNormalize function and scaled using the ScaleData function. PCA was performed utilizing the top 2000 highly variable genes recognized by the function FindVariableGenes. Batch effect removal was conducted using the package harmony (v1.0.3).

### 4.6 Cell clustering and annotation

Cell clustering was conducted based on the top 20 principal components using the graph‐based clustering algorithm in the FindClusters function with a resolution of .5. For visualization, UMAP was executed on the first 20 principal components via Seurat's RunUMAP function. Highly DEGs within a certain cluster were identified by comparison with all other clusters using the Wilcoxon test in the FindAllMarkers function (min.pct = .25, only.pos = *T*, logfc.threshold = .25 and *p*.adjust.method = ‘BH’; Table S9). Clusters obtained from the initial round of clustering were annotated according to the expression patterns of canonical marker genes (Figure S2B). Clusters showing heterogeneous expression of established marker genes were isolated, and a second round of dimensionality reduction and clustering was performed on filtered clusters (recalculation of UMAP and graph‐based clustering; this was mainly utilized to separate T cells and NK cells, myeloid cells and B cells). Clusters co‐expressing canonical markers of multiple major cell types were deemed likely doublets or low‐quality cells and omitted from further analysis. Following the removal of low‐quality cells, the refined populations included 27 359 CD4^+^ T cells, 23 465 CD8^+^ T cells, 13 632 NK cells, 18 808 myeloid cells and 13 299 B cells for subsequent analyses.

### 4.7 Single‐cell RNA‐seq signature score

Signature scores were calculated for T cells and monocytes to further characterize cell‐type‐specific subsets. We utilized published signature gene lists for cytotoxicity, exhaustion, Treg, monocyte and MDSC (Table S10).^87^ The AddModuleScore function in the Seurat package was employed to estimate the average expression of these gene signatures. To evaluate the functional variations among NK cell subsets, we defined cytotoxicity, inflammatory and stress‐related gene sets based on the previous study.^88^ The detailed composition of each gene set is provided in Table S10. NK cell scores were calculated using the AUCell package (v1.20.1).

### 4.8 Ingenuity pathway analysis

DEGs between ASD and TD groups in various subsets were determined using the FindAllMarkers function with default settings in Seurat (Table S11). Pathway enrichment was performed using IPA software (version 42012434; Ingenuity Systems; Qiagen China Co., Ltd.).

### 4.9 Cell–cell interaction analysis

We utilized the CellChat package (v1.6.1)^89^ to quantitatively infer and analyse intercellular communication networks between monocytes and T/NK cells. The interaction strength and counts between these two cell types were calculated based on the differential expression of ligand and receptor gene pairs. The two‐tailed Wilcoxon rank sum test was used to determine the significance of the interaction pairs at *p* < .05.

### 4.10 Metabolic pathway activity analysis

For each PBMC lineage, the activity of selected metabolic pathways was evaluated following previously described methods.^90^ Briefly, we computed the average expression levels of genes belonging to metabolic pathways within each cell lineage. Subsequently, mean expression levels were normalized to the overall mean values for these genes across all cell lineages. Pathway activity score significance was tested using 1000 random permutations of cell cluster assignments, with scores recomputed for each permutation. Metabolic pathways, along with their associated gene sets for specific metabolic processes, were defined using the KEGG (downloaded from KEGG BRITE: KEGG Orthology (KO)).

### 4.11 Data visualization

The scRNA‐seq data were visualized using Featureplot function in Seurat; dot plots, violin plots and stacked bar plots were created using the package ggplot2 (v3.4.3) in R; DEGs were visualized using jjVolcano function in package scRNAtoolVis (v0.0.7); and heatmap plots were generated with Complexheatmap package (v2.14.0).

### 4.12 Bulk RNA sequencing

Total RNA was extracted with TRIzol reagent (Invitrogen) and quantified using a NanoDrop ND‐1000 spectrophotometer (NanoDrop). RNA integrity was assessed with a Bioanalyzer 2100 (Agilent), confirmed by RIN > 7.0 and further verified by denaturing agarose gel electrophoresis. From 1 µg total RNA, poly(A) RNA was purified twice with Dynabeads Oligo(dT)25 (Thermo Fisher) and fragmented using the Magnesium RNA Fragmentation Module (NEB) at 94°C for 5–7 min. First‐strand cDNA was synthesized with SuperScript II Reverse Transcriptase (Invitrogen) and second‐strand cDNA with E. coli DNA polymerase I, RNase H and dUTP (NEB). After A‐tailing, fragments were ligated to indexed adapters, size‐selected with AMPureXP beads and treated with UDG (NEB). Libraries were PCR‐amplified for eight cycles, starting with 95°C for 3 min. The final cDNA library (average insert 300 ± 50 bp) was sequenced on an Illumina NovaSeq 6000 (LC‐Bio Technology) in 2 × 150 bp paired‐end mode.

### 4.13 Processing and analysis of bulk RNA‐seq data

The raw FASTQ files were processed with Trim Galore (v0.6.10), which incorporates Cutadapt and FastQC for adapter removal and quality filtering. Clean reads were retained for downstream analysis. The reference genome index for Homo sapiens GRCh38 was built, and paired‐end reads were aligned using HISAT2 (v2.2.1).^91^ Gene‐level read counts were obtained with featureCounts (v2.0.6),^92^ and mRNA expression was quantified as FPKM using StringTie (v2.1.0). The count matrix was input into DESeq2 (v1.38.1).^93^ Only genes detected in over 75% of cells were retained. DEGs between the ASD and TD groups were identified using the DESeq function, with the criteria log2 (fold change) > 1.5 and an adjusted *p* < .05 (adjusted by the Benjamini–Hochberg method). To calculate the expression of DEGs found in both bulk RNA‐seq and scRNA‐seq data, we extracted the expression profile of each cluster from the integrated slot of a Seurat object by the function AverageExpression. Heatmaps displaying the expression of significantly upregulated and downregulated genes were generated using ComplexHeatmap (v2.14.0). To evaluate the immune cell proportions in each sample, we used the ImmuneCellAI database^26^ to infer the relative abundance of 24 immune cells among PBMCs, with the FPKM gene matrix as input. The two‐tailed Wilcoxon test was used to compare the differences between ASD and TD groups.

### 4.14 Olink analysis for plasma proteomics

Protein concentrations were quantified using the Olink protein biomarker panel (Olink Proteomics AB), according to the supplier's protocol. Proximity extension assay technology^94^ enables simultaneous quantification of 92 analytes from 1 µL of sample. In this approach, paired antibody probes carrying oligonucleotides bind to target proteins, and their proximity allows oligonucleotide hybridization. DNA polymerase then initiates proximity‐dependent DNA synthesis, generating a unique PCR target sequence for detection on a Biomark HD microfluidic qPCR system (Fluidigm). Internal extension and inter‐plate controls were applied for quality control and normalization. Protein quantification is expressed as normalized expression values in log_2_ units, in which elevated values signify increased abundance. Assay validation details, including sensitivity and precision, are available from the manufacturer (www.olink.com). Additionally, KEGG enrichment analysis of significantly increased proteins in ASD was performed using the R package clusterProfiler (v4.6.0), and the gene co‐expression network was constructed using the R package WGCNA (v1.71).

### 4.15 LC–MS analysis for the plasma metabolome

Metabolites were extracted from thawed plasma with a 50% methanol buffer. In brief, 20 µL of sample was mixed with 120‐µL pre‐chilled methanol, vortexed, incubated 10 min at RT and stored overnight at −20°C. After centrifugation (4000 *g*, 20 min), supernatants were stored at −80°C for LC–MS. QC pools were made by mixing 10 µL from each extract. LC–MS was performed on a SCIEX UPLC with an ACQUITY T3 column (35°C, .4 mL/min) using a 5%–100% B gradient over 7 min. Injection volume was 4 µL. A TripleTOF 5600plus spectrometer (positive/negative modes) acquired data from 60 to 1200 Da in IDA mode, with survey scans (150 ms) and up to 12 product ion scans above 100 cps. Cycle time was .56 s, with dynamic exclusion of 4 s. QC samples were run periodically to monitor stability. Metabolites were analysed with dynamic exclusion for reliable detection and mass accuracy calibration. KEGG enrichment analysis of QMs was performed using the ClusterProfiler R package (v4.6.0).

### 4.16 Principal component analysis of multiple omics

PCA was conducted independently for mFCM, bulk RNA‐seq, metabolomic and proteomic data that met quality control standards using PCA function in package FactoMineR (v2.6). Data values were normalized to a range of 0 to 1 and mean‐centred prior to dimensionality reduction. Specifically, PCA was applied to 92 proteins per individual, 5491 metabolites, all proportional and absolute counts in mFCM data, and all qualified genes in bulk RNA‐seq. Results were visualized using the fviz_pca_ind function.

### 4.17 NK cell cytotoxicity assay

Co‐culture assay was assessed by co‐incubation of NK cells with K562 cells for 4 h. Cytolytic capacity was evaluated based on the ability to induce apoptosis in K562 cells, measured using the Annexin V apoptosis kit (BD, Cat# 559763) and analysed through flow cytometry.

### 4.18 LDH cytotoxicity assay

LDH cytotoxicity assay was conducted with a commercial LDH Cytotoxicity Assay Kit (Abcam, Cat#ab65393) following the supplier's protocol. K562 cells (2 × 10^4^ cells/well) were plated in 48‐well plates and incubated at 37°C for 24 h. NK cells (2 × 10^5^ cells/well) were then added to the wells for co‐culture. At 0, 2, 4 and 6 h after incubation at 37°C, 10 µL of supernatant was taken from each well and mixed with 100 µL LDH reaction mix. Following a 30 min incubation at RT, absorbance was determined at 450 nm.

### 4.19 Statistical analysis

Data are presented as the mean ± standard deviation. For normally distributed variables, two‐tailed unpaired *t*‐tests or paired *t*‐tests were employed to compare the two groups. For non‐normally distributed variables, two‐tailed Wilcoxon rank sum tests were used to applied. The Benjamini–Hochberg method was utilized to correct for multiple testing. Pearson correlation was used for continuous and normally distributed variables, whereas Spearman correlation was employed for non‐continuous data. The correlation coefficients were computed using the R package Hmisc (v4.2.7), with significance reported as *p* values calculated by the cor function in R package stats (v4.2.2). *p* values < .05 were considered statistically significant. For analyses conducted using GraphPad Prism (v.10.1.0), significance is expressed as a *p* value. For analyses performed by R (v4.1.3), significance levels are indicated by asterisks: ^*^ *p* < .05; ^**^ *p* < .01; ^***^ *p* < .001; and ^****^ *p* < .0001.

## AUTHOR CONTRIBUTIONS

Yufeng Liu, Wenxiong Chen, Yuxin Wu and Chun Yan designed experiments. Yufeng Liu, Wenxiong Chen, Chun Yan, Fangmei Feng and Chaoting Lan performed the experiments. Yufeng Liu, Wenxiong Chen, Yuxin Wu, Chun Yan, Chaoting Lan, Xiaotao Jiang, Huijuan Wang, Yinchun Chen, Yuling Yang, Xiaoli Huang and Gang Luo analysed data. Yufeng Liu, Wenxiong Chen and Chun Yan wrote the manuscript, with all authors contributing to writing and providing feedback. Yufeng Liu, Chun Yan, Liangqiong Deng and Chaoting Lan revised the manuscript.

## CONFLICT OF INTEREST STATEMENT

The authors declare no conflicts of interest.

## ETHICS STATEMENT

This study was approved by the ethics committee of Guangzhou Women and Children's Medical Center (2018031402).

## Supporting information



Supporting Information

Supporting Information

Supporting Information

Supporting Information

Supporting Information

Supporting Information

Supporting Information

Supporting Information

Supporting Information

Supporting Information

Supporting Information

Supporting Information

Supporting Information

## Data Availability

The raw sequence data reported in this paper have been deposited in the Genome Sequence Archive (Genomics, Proteomics & Bioinformatics 2021) in National Genomics Data Center (Nucleic Acids Res 2022), China National Center for Bioinformation/Beijing Institute of Genomics, Chinese Academy of Sciences (GSA‐Human: HRA007632 and HRA007654) that are publicly accessible at https://ngdc.cncb.ac.cn/gsa‐human. The remaining data are available within the paper, supplementary information or available from the authors upon request.
