# Supplementary material for: Comprehensive multi‐omics mapping of immune perturbations in autism spectrum disorder
Source: Clin Transl Med. 2025 Dec 12;15(12):e70552. doi: 10.1002/ctm2.70552 (PMC12699144; doi:10.1002/ctm2.70552)
Supplement: Supplementary file 1 — Supporting Information [file CTM2-15-e70552-s006.docx]

Supplementary Information for

**Comprehensive Multi-Omics Mapping of Immune Perturbations in Autism Spectrum Disorder**

Chun Yan^1,2,13^, Fangmei Feng^3,13^, Chaoting Lan^1,13^, Gang Luo^4^, Xiaotao Jiang^5^, Huijuan Wang^2^, Yinchun Chen^6,7^, Yuling Yang^1,2^, Liangqiong Deng^8^, Xiaoli Huang^9^, Yuxin Wu^10^*, Wenxiong Chen^11,12^*, Yufeng Liu^2,14^*

^1^ Liuzhou Hospital of Guangzhou Women and Children's Medical Center, No. 50 Boyuan Avenue, Liuzhou, Guangxi, China

^2^ Center for Medical Research on Innovation and Translation, Guangzhou First People's Hospital, the Second Affiliated Hospital of South China University of Technology, Guangzhou, Guangdong, China

^3^ Department of Neuroelectrophysiology, Guangzhou Women and Children Medical Center, Guangzhou Medical University, Guangzhou, Guangdong, China

^4^ Jianghai Street Community Health Service Center, Haizhu District, Guangzhou, Guangdong, China

^5^ First Clinical Medical College, Guangzhou University of Chinese Medicine, Guangzhou, Guangdong, China

^6^ Department of Hematology, the Second Affiliated Hospital, School of Medicine, South China University of Technology, Guangzhou, Guangdong, China

^7^ Department of Hematology, Guangzhou First People’s Hospital, Guangzhou, Guangdong, China

^8^ Department of Children Healthcare，Liuzhou Hospital of Guangzhou Women and Children's Medical Center, No. 50 Boyuan Avenue, Liuzhou, Guangxi, China

^9^ Department of Neurology, Liuzhou Hospital of Guangzhou Women and Children's Medical Center, Liuzhou Key Laboratory of Pediatric Epilepsy Prevention and Treatment, No. 50 Boyuan Avenue, Liuzhou, Guangxi, China

^10^ Department of Rehabilitation, Guangzhou Women and Children's Medical Center, Guangzhou Medical University, Guangzhou, China

^11^ Department of Neurology, Guangzhou Women and Children Medical Center, Guangzhou Medical University, Guangzhou, Guangdong, China

^12^ Department of Behavioral Development, Guangzhou Women and Children Medical Center, Guangzhou Medical University, Guangzhou, Guangdong, China

^13^ These authors contributed equally

^14^ Lead contact

^*^ Correspondence: wuyuxin.hugo@qq.com; gzchcwx@126.com; [eyyufengliu@scut.edu.cn](mailto:eyyufengliu@scut.edu.cn)

This PDF file includes:

Supplementary Figures (Figure S1 to S12)

Supplementary Figures





**Supplementary Figure 1.** **Demographic and clinical characteristics of study groups.**

**(A-B)** Boxplots illustrating the differences in age **(A)** and variations in blood routine results **(B)** between ASD patients and TD individuals. Data are presented as mean ± SD and two-tailed unpaired t tests were applied.

**(C)** Correlation matrix displaying demographic and clinical characteristics of ASD patients. Circle size corresponds to the absolute value of the Spearman correlation coefficient, with blue (red) color indicating a positive (negative) correlation. *p＜0.05, **p＜0.01, ***p＜0.001.





**Supplementary Figure 2. Characteristics of PBMC scRNA-seq data and overlapping DEGs between bulk RNA-seq and scRNA-seq profiles.**

**(A)** Violin plots depicting the distribution of feature counts, RNA counts, and mitochondrial percentages of each scRNA-seq sample post-filtering.

(**B)** UMAP plot showing the expression of canonical markers across main cell lineages by color. Each point represents one cell.

**(C)** UMAP plot illustrating the distribution of PBMCs from all individuals. Each point represents one cell, with coloring based on the sample origin.

**(D)** Bar chart showing the proportions of cell clusters among PBMCs in individual samples.

**(E)** Volcano plot visualizing DEGs in bulk RNA-seq data comparing the ASD and TD groups (criteria: adjusted p < 0.05 and absolute log2 [fold change] >1). P values were calculated via the Wald test and adjusted by the Benjamini-Hochberg method.

**(F-G)** Heatmap visualizing the row-scaled average expression of common DEGs across cell types identiﬁed in scRNA-seq data. The common DEGs refer to genes that both up-regulated/down-regulated in bulk RNA profiles and all scRNA-seq profiles of ASD patients. Upregulated DEGs are shown in (**F**) and downregulated DEGs in (**G**). Common DEGs were deﬁned as the overlap between DEGs in bulk RNA-seq data in E and DEGs in scRNA-seq data.





**Supplementary Figure 3. Gating strategy of mFCM data.**

Cla-Mono: classical-monocytes; Int-Mono: Intermediate-monocytes; Non-cla-Mono: non-classical-monocytes; MDSCs: myeloid-derived suppressor cells; PMN-MDSCs: polymorphonuclear-MDSCs; M-MDSCs: monocytic-MDSCs; e-MDSCs: early-MDSCs; T_N_: T naïve; T_CM_: T central memory ; T_EM1/2/3_: T effector memory 1/2/3; T_EMRA_: T effector memory re-expressing CD45RA; Act CD4^+^ T/ CD8^+^ T：activated CD4^+^ T or CD8^+^ T cells; NK^Dim^, conventional CD56^dim^CD16^pos^ NK cells; NK^Uc^, unconventional CD56^dim^CD16^neg^ NK cells; NK^Bri^, conventional CD56^bright^CD16^neg^ NK cells.





**Supplementary Figure 4. Additional details on T cells, related to Figure 2.**

**(A)** Proportions and absolute counts of activated CD4^+^ T and CD8^+^ T cells in ASD patients (n=14) and TD individuals (n=34). Data are presented as mean ± SD and two-tailed unpaired t tests were applied.

(**B)** Representative ﬂow cytometry gating plot for Tregs.

**(C-E)** Representative ﬂow cytometry gating plot for Th cells (**C**), Tph, Tfh, pro-Tph (**D**), and Tph subsets (**E**).

**(F)** UMAP plot illustrating the distribution of CD4^+^ T and CD8^+^ T cells from all individuals. Each point represents one cell, colored according to sample origin (left) and group classification (right).

**(G)** Heatmap representing the scaled average expression values of the top 10 genes defining each T cell subsets in scRNA-seq profiles.

**(H)** Heatmap showing the scaled average expression values of T cell functional markers. The color represents the average expression.

**(I)** Combined dot plot representing the DEGs between ASD and TD individuals across T cell subsets in scRNA-seq profiles, with red (blue) color indicating elevated (decreased) expression in ASD.





**Supplementary Figure 5. IPA results of DEGs between ASD and TD groups in T cell subsets.**

The ingenuity pathway analysis (IPA) of DEGs between ASD and TD scRNA-seq profiles in selected T cell subsets. The left panel represents the graphical summaries and the right panel represents bar plots of the ingenuity canonical pathways enriched, with orange (blue) color indicating predicted activation (inhibition) in ASD.


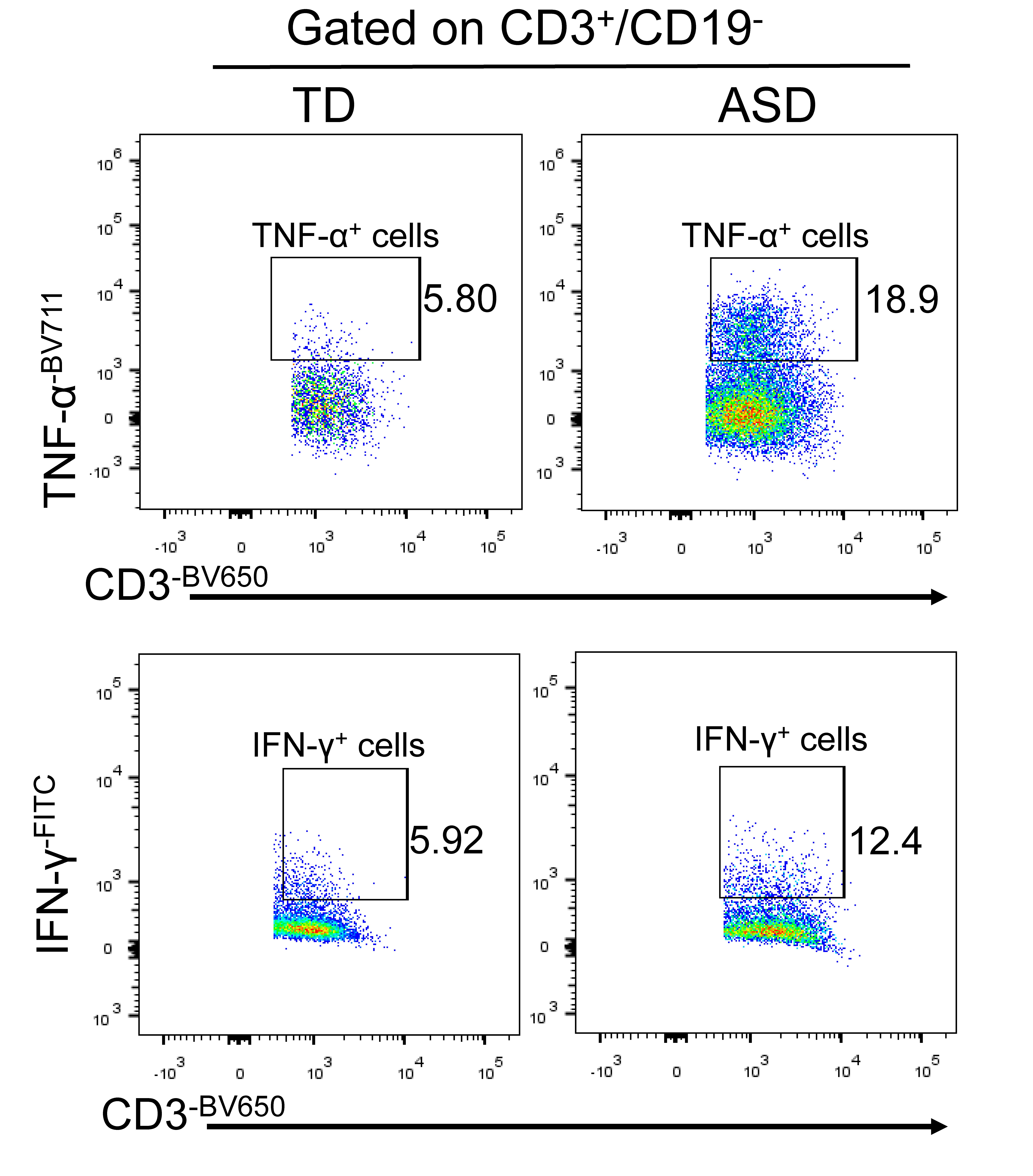


**Supplementary Figure 6. Representative ﬂow cytometry gating plot for IFN-γ^+^ T cells and TNF-α^+^ T cells.**



**Supplementary Figure 7. Additional details on NK cells, related to Figure 3.**

**(A)** Comparison of NK cell cytotoxicity between ASD and TD samples (n=5 per group) at 0, 2, 4, and 6 hours, measured by LDH release assay. Data are presented as mean ± SD. Statistical significance was determined using unpaired t-tests; p-values are indicated above the bars.

**(B)** Heatmap representing the scaled average expression values of the top 10 genes defining each NK cell subsets in scRNA-seq profiles. The color gradient represents the average expression levels across subsets.

**(C)** UMAP plot showing the expression of representative markers of NK subsets by color. Each point represents one cell.

**(D)** Combined dot plot representing the DEGs between ASD and TD individuals across NK cell subsets in scRNA-seq profiles, with red (blue) color indicating elevated (decreased) expression in ASD.





**Supplementary Figure 8. IPA results of DEGs between ASD and TD groups in NK cell subsets.**

The ingenuity pathway analysis (IPA) of DEGs between ASD and TD scRNA-seq profiles in selected NK cell subsets. The left panel represents the graphical summaries and the right panel represents bar plots of the ingenuity canonical pathways enriched, with orange (blue) color indicating predicted activation (inhibition) in ASD.





**Supplementary Figure 9. Additional details on myeloid cells, related to Figure 4.**

**(A)** Proportion of non-T/B cells in ASD patients (n=43) and TD individuals (n=46). Data are presented as mean ± SD and two-tailed unpaired t tests were applied.

**(B-C)** Heatmap and Dot plot representing the expression values of the highly expressed genes defining each myeloid subset in scRNA-seq profiles. The size of each circle in (**C**) corresponds to the percentage of cells in the subtype expressing the gene, and the color represents the average expression.

**(D)** Dot plot showing the average expression levels and the percentage of cells expressing monocyte and MDSC signature scores in monocyte subsets from all scRNA-seq samples.

(**E)** Bar plot illustrating cell abundances across myeloid subsets for ASD and TD groups.

(**F)** Dot plot comparing the signiﬁcant ligand-receptor pairs between ASD and TD groups, which contribute to the signaling from Mono_c03_FCGR3A to NK and T cell subsets. The highlighted TNF-TNFRSF1B, TNF-TNFRSF1B, and CXCL10-CXCR3 signaling were up-regulated in ASD group. Dot color reﬂects communication probabilities and dot size represents computed p-values. Empty space means the communication probability is zero. P-values are computed from one-sided permutation test.

(**G)** Combined dot plot representing the DEGs between ASD and TD scRNA-seq profiles across monocyte subsets, with red (blue) color indicating elevated (decreased) expression in ASD.





**Supplementary Figure 10. IPA results of DEGs between ASD and TD groups in monocyte subsets.**

The ingenuity pathway analysis (IPA) of DEGs between ASD and TD scRNA-seq profiles in monocyte subsets. The left panel represents the graphical summaries and the right panel represents bar plots of the ingenuity canonical pathways enriched, with orange (blue) color indicating predicted activation (inhibition) in ASD.





**Supplementary Figure 11. Additional details on plasma metabolites, related to Figure 6.**

**(A)** Heatmap illustrating the differential metabolites identified between the ASD and TD groups.

**(B)** The numbers of differential positive metabolites and negative metabolites.

**(C)** Heatmap visualizing the metabolic pathway activity scores of six major cell lineages in scRNA-seq profiles. The red box indicated the nucleotide metabolism, energy metabolism, and lipid metabolism pathways were highlighted.

**(D-E)** Correlation matrix between plasma differential metabolites (**D**: up-regulated in ASD; **E**: down-regulated in ASD) and clinical diagnostic scales scores. The circle size corresponds to the absolute value of the Spearman correlation coefficient, with red (blue) color indicating a positive (negative) correlation. *p＜0.05, **p＜0.01, ***p＜0.001.





**Supplementary Figure 12. Characteristics of B cells in ASD patients.**

**(A)** UMAP embedding of 13,299 B cells from all scRNA-seq samples. Each point represents one cell. Cells colored according to the cell cluster.

(**B-C)** Heatmap and Dot plot representing the expression values of the highly expressed genes defining each B subsets in scRNA-seq profiles. The size of each circle in (**C**) corresponds to the percentage of cells in the subtype expressing the gene, and the color represents the average expression.

**(D)** Bar plot highlighting cell abundances across B subsets for ASD and TD groups. **e** Representative ﬂow cytometry gating plot for B subsets.

**(F)** Proportions and absolute counts of B cell subsets in ASD patients (n=23-44) and TD individuals (n=11-40). Data are presented as mean ± SD and two-tailed unpaired t tests were applied.

(**G)** Combined dotplot representing the DEGs between ASD and TD scRNA-seq profiles across B subsets, with red (blue) color indicating elevated (decreased) expression in ASD.





**Supplementary Figure 13. IPA results of DEGs between ASD and TD groups in B cell subsets.**

The ingenuity pathway analysis (IPA) of DEGs between ASD and TD scRNA-seq profiles in selected B subsets. The left panel represents the graphical summaries and the right panel represents bar plots of the ingenuity canonical pathways enriched, with orange (blue) color indicating predicted activation (inhibition) in ASD.
