# Supplementary material for: Comprehensive multi‐omics mapping of immune perturbations in autism spectrum disorder
Source: Clin Transl Med. 2025 Dec 12;15(12):e70552. doi: 10.1002/ctm2.70552 (PMC12699144; doi:10.1002/ctm2.70552)
Supplement: Supplementary file 2 — Supporting Information [file CTM2-15-e70552-s004.docx]

File Name: Supplementary Table 1

Description: Characteristics and ASD rating scale scores of ASD patients and TD individuals.

File Name: Supplementary Table 2

Description: Comparison of demographic characteristics between ASD patients and TD individuals.

File Name: Supplementary Table 3

Description: Blood routine test results of ASD patients and TD individuals.

File Name: Supplementary Table 4

Description: Differentially expressed genes of bulk RNA-seq profiles between two groups.

File Name: Supplementary Table 5

Description: Cell proportions and absolute counts of each immune cell type analyzed by mFCM.

File Name: Supplementary Table 6

Description: mFCM antibody information.

File Name: Supplementary Table 7

Description: Results of plasma proteomics

File Name: Supplementary Table 8

Description: Results of plasma metabolome

File Name: Supplementary Table 9

Description: Highly differentially expressed genes of each subset in scRNA-seq profiles.

File Name: Supplementary Table 10

Description: The gene signatures for monocytes, MDSCs, naïve T cells, Tregs, T cell exhaustion, and cytotoxic T cells, along with the NK cell gene sets.

File Name: Supplementary Table 11

Description: Differentially expressed genes identified in each subset of scRNA-seq profiles between two groups
